# Pan-cancer noncoding genomic analysis identifies functional *CDC20* promoter mutation hotspots

**DOI:** 10.1016/j.isci.2021.102285

**Published:** 2021-03-09

**Authors:** Zaoke He, Tao Wu, Shixiang Wang, Jing Zhang, Xiaoqin Sun, Ziyu Tao, Xiangyu Zhao, Huimin Li, Kai Wu, Xue-Song Liu

**Affiliations:** 1 School of Life Science and Technology, ShanghaiTech University, Shanghai 201203, China; 2 Department of Thoracic Surgery, The First Affiliated Hospital of Zhengzhou University, Zhengzhou 450052, China; 3 Shanghai Institute of Biochemistry and Cell Biology, Chinese Academy of Sciences, Shanghai, China; 4 University of Chinese Academy of Sciences, Beijing, China

**Keywords:** Genetics, Genomics, Cancer Systems Biology, Cancer

## Abstract

Noncoding DNA sequences occupy more than 98% of the human genome; however, few cancer noncoding drivers have been identified compared with cancer coding drivers, probably because cancer noncoding drivers have a distinct mutation pattern due to the distinct function of noncoding DNA. Here we performed pan-cancer whole genome mutation analysis to screen for functional noncoding mutations that influence protein factor binding. Recurrent mutations were identified in the promoter of *CDC20* gene. These *CDC20* promoter hotspot mutations disrupt the binding of ELK4 transcription repressor, lead to the up-regulation of *CDC20* transcription. Physiologically ELK4 binds to the unmutated hotspot sites and is involved in DNA damage-induced *CDC20* transcriptional repression. Overall, our study not only identifies a detailed mechanism for *CDC20* gene deregulation in human cancers but also finds functional noncoding genetic alterations, with implications for the further development of function-based noncoding driver discovery pipelines.

## Highlights

• Pan-cancer noncoding analysis for mutations that influence protein factor binding
• Recurrent mutations were identified in the promoter of *CDC20* gene
• Promoter hotspot mutations disrupt ELK4 binding, up-regulate *CDC20* transcription
• Promoter hotspot mutation site is involved in DNA damage-induced *CDC20* repression

## Graphical abstract

**Figure:**
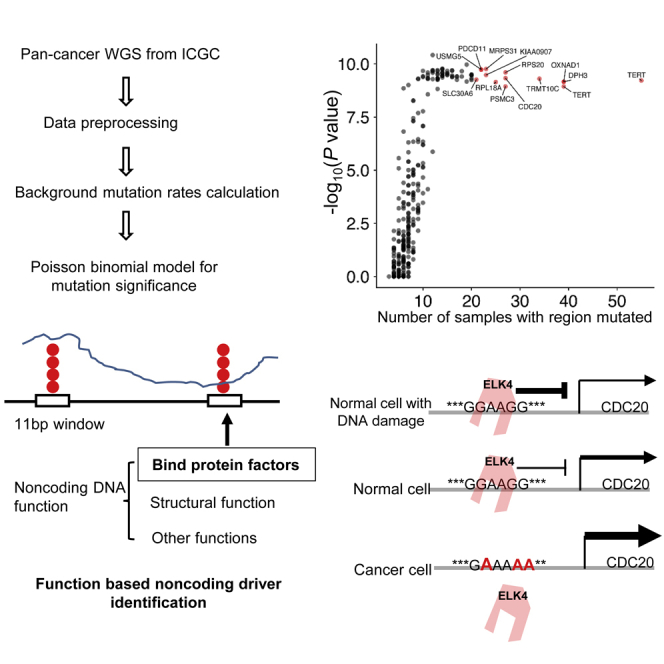

## Introduction

Cancer develops primarily because of somatic alterations in the genomic DNA. Somatic mutations in noncoding sequences are poorly explored in cancer, a rare exception being the recent identification of *TERT* promoter mutations (Bell et al., 2015; Horn et al., 2013; Huang et al., 2013). Recently, there have been several research efforts in identifying significantly mutated noncoding sites (Fredriksson et al., 2014; Lochovsky et al., 2015; Melton et al., 2015; Rheinbay et al., 2017; Weinhold et al., 2014; Zhang et al., 2018). Weinhold et al. performed whole-genome sequences (WGS) analysis of 863 pan-cancer samples. Besides *TERT* promoter, some other recurrent promoter mutation hotspots were identified, such as *PLEKHS1*, *WDR74,* and *SDHD* (Weinhold et al., 2014). Fredriksson et al. analyzed 505 tumor genomes across 14 cancer types and identified no other frequent oncogenic promoter mutations beyond *TERT*. It was thus speculated that *TERT* promoter mutation is a rare exception in searching for cancer-driving noncoding genetic alterations (Fredriksson et al., 2014). A recent pan-cancer analysis of whole genomes (PCAWG) study with 2,658 WGS samples also suggested that noncoding drivers are rare compared with protein-coding drivers (Rheinbay et al., 2020).

It has been predicted by the Encyclopedia of DNA Elements (ENCODE) project that roughly 80% of the human genome has biological function (Consortium, 2012). Somatic mutations in noncoding regions are frequent. Disease-associated genomic variations are also frequently located in noncoding regions (Maurano et al., 2012). It is reasonable to expect that cancer should have a substantial number of noncoding driver genetic alterations. However, currently only a few cancer-driving noncoding genetic alterations have been identified, probably because of the following reasons. First, the mutation patterns of noncoding drivers are different from the mutation patterns of coding drivers. Noncoding DNA could have distinct functions: some may code noncoding RNA, some may have structural function, and some may function by binding protein factors. And this is different from coding regions, which function through coding proteins. Consequently, cancer noncoding drivers could have distinct mutation patterns compared with coding drivers, thus requiring distinct methods to identify these noncoding drivers. Second, an insufficient number of patients have been sequenced to identify significantly mutated noncoding elements, especially for those noncoding drivers that occurred at low frequency. Third, there is low sequencing coverage in noncoding regions. Owing to sequencing cost, exome sequencing is preferred over WGS in many cancer genomics studies, and noncoding DNA are not covered in these cancer genomic studies. Furthermore, noncoding sequences, especially those that are GC rich or contain repetitive sequences have especially low sequence coverage in second-generation WGS (Rheinbay et al., 2017).

Here we have used so far the largest number of WGS samples to systematically screen for potentially cancer-driving noncoding DNA mutations. Our analysis emphasizes the protein binding function of noncoding sequences. We recapitulated well-known noncoding drivers, such as *TERT* promoter mutations. In addition, we identified novel promoter mutation hotspots in *CDC20*, which is a known cancer-related gene. Further experimental studies supported an oncogenic function of these *CDC20* promoter mutations.

## Results

### Noncoding mutation analysis of human cancer genome

**Figure 1:**
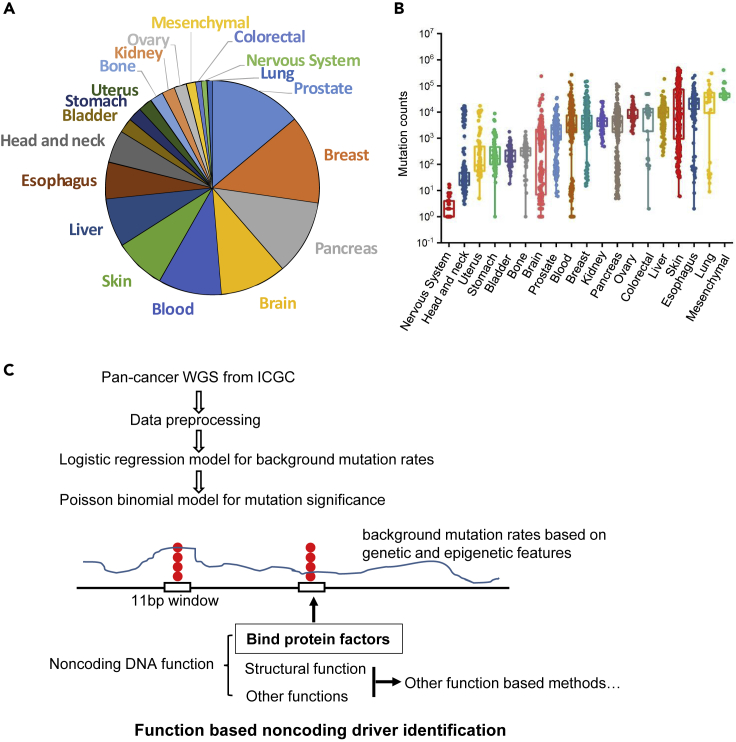
Summary of pan-cancer noncoding analysis data and workflow (A) Proportion of tumor samples by disease types. (B) Mutation count distribution of individual samples in 19 cancer types. (C) Workflow of the method to detect recurrently mutated noncoding regions that affect protein factor binding.

To obtain the most mutations in genome noncoding regions, we selected patients with tumor with WGS data, filtered out donors with hyper-mutations, and chose single-nucleotide alteration (point mutation) as the focus of this study. Mutations that were potentially false-positive from mapping errors or represented common single-nucleotide polymorphisms were removed from further analysis. After filtering, WGS data of 4,859 donors from 19 cancer types have been included in this study (Figures 1A and S1). The average mutation count for the overall sample is 9,819, and in total 47,708,263 mutations have been included in this study. The distribution of mutation counts in each sample is shown, and most samples have mutation counts less than 20,000 (Figure 1B). There are big differences in mutation burdens between cancer types or between samples with the same cancer type (Figure 1B).

To identify the factors that influence background mutation rates, we performed correlation analysis between genetic and epigenetic features with background mutation rates. It has been reported that mutation rates in cancer genomes are highly correlated with chromatin organization status, and the arrangement of the genome into heterochromatin- and euchromatin-like domains is a dominant influence on regional mutation-rate variation in human somatic cells (Schuster-Bockler and Lehner, 2012). Here we analyzed the correlations between genetic or epigenetic features and mutation rates in coding and noncoding regions (Figure S2A). The following genetic features have been included in this analysis: genome mappability, replication timing, transcription factor binding sites (TFBS), GC content, CpG island, DNA polymerase II, DNA conservation, and recombination rate. The following epigenetic features were also included: DNase I hypersensitive site and histone modifications (H3K4me1, H3K4me3, H3K27me3, H3K36me3, H3K9me3, H3K27ac, and H3K9ac). We then calculated the correlation coefficients for all genetic or epigenetic features with background mutation rates and found that at the megabase scale, cancer noncoding mutation rates show strong correlation with several features of chromatin structure (Figure S2B). Heterochromatin markers H3K27me3 and H3K9me3 are associated with increased noncoding mutation rates (Figure S2B). TFBS show elevated mutation rates (Mao et al., 2018) (Figure S2D). Furthermore, these correlations in noncoding regions are similar to the correlations in coding regions (Figures S2B and S2C), suggesting that the background mutation rates in both coding and noncoding regions are similarly influenced by these genetic or epigenetic features.

### Pan-cancer genomic analysis to identify noncoding mutation hotspots

To identify positive selection in cancer genomes, it is essential to build an accurate background mutation rate model that corrects for covariates (features) that impact regional mutation rate variation, such as local sequence context and chromatin features (Schuster-Bockler and Lehner, 2012). Our algorithm employed logistic regression to determine sample-specific and covariate-corrected background mutation probabilities followed by a Poisson binomial model to account for patient-specific probabilities (Figures 1C and S3). Logistic regression was performed to calculate the expected probability (or background probability) for each genome site. We considered a range of genetic and epigenetic features that correlated with somatic noncoding mutation rates, including genetic features (sequence context, replication timing, TFBS, conservation, GC content, CpG density, promoter) and epigenetic features (DNase I hypersensitive site and histone modifications H3K4me1, H3K4me3, H3K27me3, H3K36me3, H3K9me3, H3K27ac, and H3K9ac).

Non-protein-coding DNA elements could have the following potential functions: code for non-protein-coding RNA, act as *cis*-regulatory elements, or serve for some unknown structural function. The *cis*-regulatory elements include proximal regulatory elements (promoters, etc.) and distal elements (enhancers, silencers, insulators, etc.). Most of these *cis*-regulatory noncoding DNA elements function through binding protein factors. Here we developed an analysis framework that emphasized the protein binding function of noncoding DNA sequences (Figure 1C). To identify noncoding mutations that could have potentially functional consequence in protein binding, we focused on clustered mutation hotspots. As most protein factors bind DNA 6–10 bp long, the clustered regions were defined as a 10-bp DNA surrounding the recurrently mutated sites. The probability that mutation happened in this 11-bp window was calculated with a Poisson binomial distribution model. Noncoding mutations in promoter regions (within 5 kb of gene transcription start sites) were further selected in downstream analysis and experimental validation.

**Figure 2:**
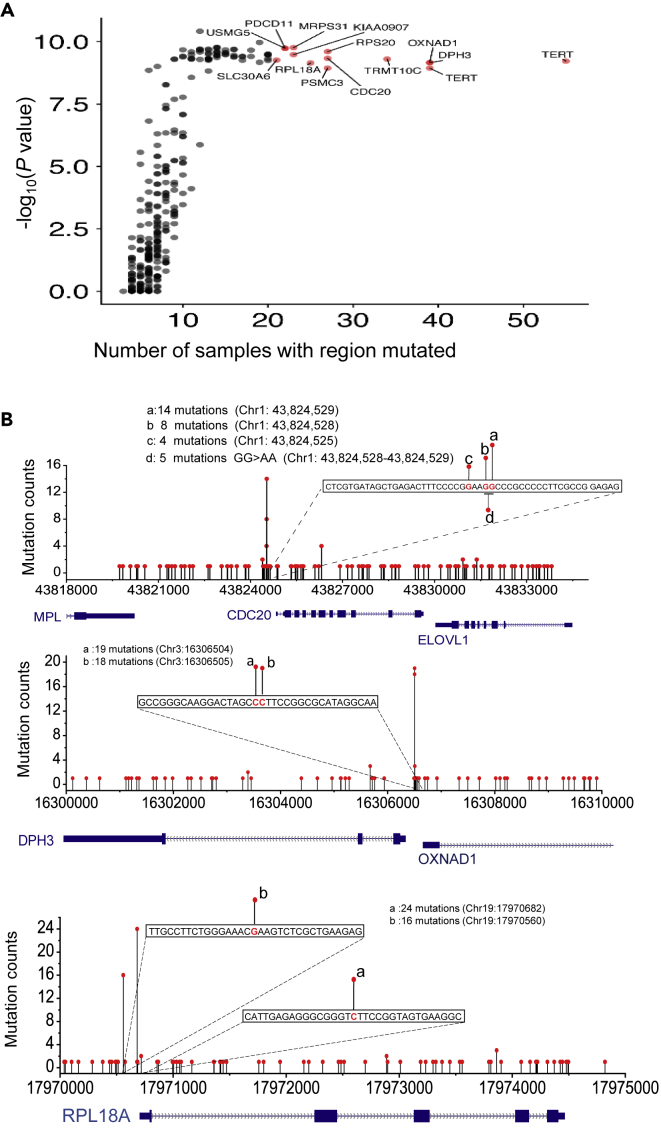
Noncoding mutation hotspots analysis (A) Shown is the probability (−log_10_) of noncoding mutations in the 11-bp window (y axis) plotted against the number of times the noncoding region is found mutated (x axis). Bonferroni-adjusted p values are shown. (B) Typical noncoding mutation hotspots in regulatory regions of *CDC20*, *DPH3*/*OXNAD1,* and *RPL18A* genes are shown.

We ranked the selected 11-bp noncoding regions based on calculated mutation probability and mutation frequency (Figure 2A), and *TERT* promoter mutations are top ranked (Figures2A and S4). Some of the previously reported significantly mutated promoters were identified, such as *DPH3* promoter mutations (Denisova et al., 2015) (Figure 2B). In addition, some novel noncoding mutation hotspots were also identified, including promoter mutations of *RPL18A* (Figure 2B). Patients with melanoma with hotspot mutations in *RPL18A* promoter have significantly poorer prognoses compared with patients without those hotspot mutations (Figure S5). The function of most of these identified noncoding mutations is unknown. Interestingly, we identified novel recurrent clustered mutations in the promoter region of *CDC20* gene (Figure 2B and Table S1). Similar analyses were performed with the selection of different window sizes from 7 to 21 bp, and *CDC20* promoter mutations are top ranked in all these analyses (Figure S6). To identify mutational clusters in noncoding regions in liver cancer, Fujimoto et al. selected a 500-bp window to calculate the statistical significance (Fujimoto et al., 2016). The significantly mutated regions identified with these larger windows may not directly influence the binding of protein factors. Recurrent indels in the promoter regions are shown (Figure S7), and clustered mutations in 3′-UTR, 5′-UTR, and intron regions are also shown (Figure S8).

### Genetic alterations of *CDC20* in human cancers

CDC20 was discovered in the early 1970s when Hartwell et al. made yeast mutants that failed to complete cell cycle progression (Hartwell et al., 1970). The *CDC20* mutant could not enter anaphase (Hartwell et al., 1973). In 1995, the biochemical function of *CDC20* became clear after the discovery of the APC/C (King et al., 1995; Sudakin et al., 1995). The APC/C-CDC20 protein complex plays a key role in cell cycle spindle checkpoint and metaphase-to-anaphase transition mainly through two protein targets. First, it targets securin for destruction, enabling the eventual destruction of cohesin and thus sister chromatid separation. It also targets cyclins for destruction, which inactivates cyclin-dependent kinases (Cdks) and allows the cell to exit from mitosis (Pesin and Orr-Weaver, 2008).

**Figure 3:**
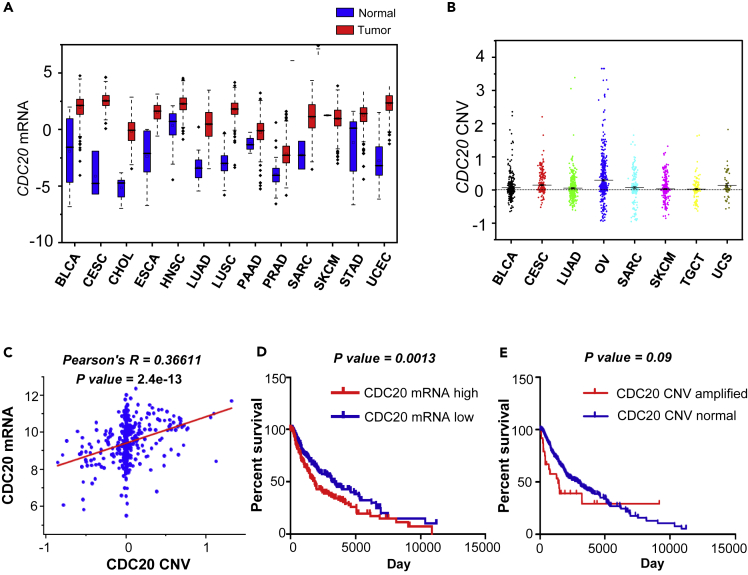
mRNA and CNV analysis of *CDC20* in various human cancers (A) *CDC20* mRNA expression levels were compared in multiple types of human cancers and corresponding normal control tissues based on The Cancer Genome Atlas (TCGA) database. The boxplot is bounded by the first and third quartiles with a horizontal line at the median. (B) *CDC20* CNV levels in various cancers are shown based on TCGA datasets. The unit is Gistic2 copy number. (C) The correlation between *CDC20* CNV and mRNA in TCGA melanoma samples (n = 367). Pearson correlation P and *R* values are shown. (D and E) Kaplan-Meier overall survival curves of patients with melanoma are shown. Patients are separated into two groups based on *CDC20* mRNA (D) or CNV (E) values. n = 231 for both *CDC20* mRNA high and low groups. n = 24 for *CDC20* CNV amplified group and n = 333 for CDC20 CNV normal group. Log rank (Mantel-Cox) test p values are shown. BLCA: bladder cancer; CESC: cervical cancer; CHOL: bile duct cancer; ESCA: esophageal cancer; HNSC: head and neck cancer; LUAD: lung adenocarcinoma; LUSC: lung squamous cell carcinoma; PAAD: pancreatic cancer; PRAD: prostate cancer; SARC: sarcoma; SKCM: melanoma; STAD: stomach cancer; UCEC: endometrioid cancer. OV: ovarian cancer; TGCT: testicular germ cell tumors; UCS: uterine carcinosarcoma.

Previous studies reported that *CDC20* is overexpressed in various human cancers (Chang et al., 2012; Gayyed et al., 2016; Kim et al., 2014; Wang et al., 2013). We systematically compared the mRNA expression of *CDC20* between cancer and normal tissues in various cancers based on TCGA datasets. In nearly all types of cancers analyzed, elevation of *CDC20* mRNA expression is observed (Figure 3A). These data validated previous observations. Recurrent genetic alterations are typical features of cancer-driving genes. We further analyzed genetic alterations in *CDC20* genes based on public cancer genome databases. No recurrent somatic mutations in *CDC20* coding sequence were identified. However, the copy number variation (CNV) of *CDC20* shows amplification in various cancers including ovarian cancer, bladder cancer, cervical cancer, etc (Figure 3B). *CDC20* CNV shows significant positive correlation with *CDC20* mRNA (Figure 3C). Genetic amplification of *CDC20* suggests an oncogenic driving function of *CDC20* in cancer progression.

It has been reported that overexpression of *CDC20* promoted cancer progression, whereas its knockdown suppressed cancer (Majumder et al., 2014; Mukherjee et al., 2013). *CDC20* was suggested as a legitimate target of drug development for the treatment of human malignancies (Wang et al., 2013). We studied the prognosis of *CDC20* mRNA expression in melanoma. As previously reported, *CDC20* mRNA overexpression leads to significantly poorer melanoma prognosis (Figure 3D). *CDC20* CNV amplification also tends to result in poorer melanoma prognosis (Figure 3E). Taken together, these data support an oncogenic driving function of *CDC20* in human cancer. The CNV amplification and mRNA up-regulation of *CDC20* in cancer versus normal is one rationale for us to further investigate the function of these *CDC20* promoter noncoding hotspot mutations.

### Recurrent promoter mutations stimulate *CDC20* transcription

**Figure 4:**
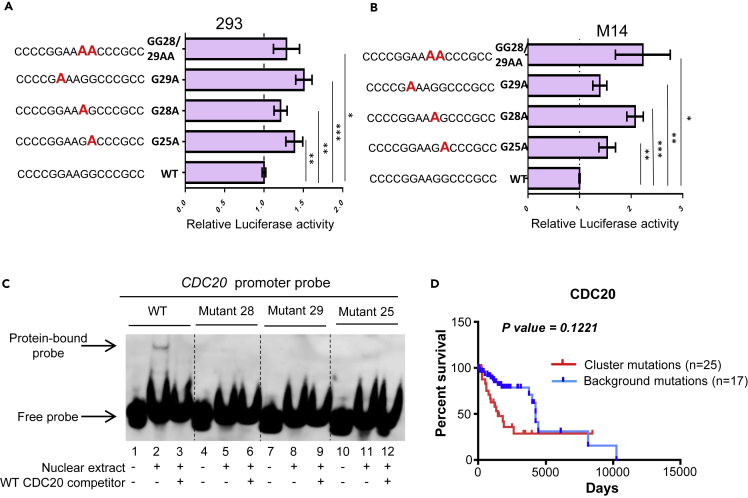
Functional consequence of *CDC20* promoter hotspot mutations (A and B) Luciferase reporter assay was performed in 293 (A) and M14 cells (B) with wild-type (WT) or mutant *CDC20* promoter driving luciferase vectors. Error bars represent mean ± SD from three experiments. ∗p < 0.05, ∗∗p < 0.01, ∗∗∗p < 0.001. Unpaired Student's t test p values between each mutation and WT control are shown. (C) EMSA assays were performed with wild-type or mutant *CDC20* promoter probes. *CDC20* promoter mutation strongly abolish protein factor binding to DNA probes. (D) Kaplan-Meier overall survival curves of patients with melanoma with indicated *CDC20* promoter mutations or control mutations. n = 25 for patients with clustered *CDC20* promoter mutations (including G25A, G28A, G29A, and GG28/29AA), n = 17 for patients with other mutations in the background region. Log rank (Mantel-Cox) test p value is shown.

To test whether the mutations identified in *CDC20* promoter region have functional consequence, we used luciferase reporter assay to evaluate the effect of each mutation on *CDC20* promoter activity. It has been reported that endogenous *CDC20* transcription can be suppressed by DNA damage drugs, such as 5-fluorouracil (5-FU) (Banerjee et al., 2009). To test if the luciferase reporter we generated can mimic the activity of endogenous *CDC20* promoter, we studied the response of our luciferase reporter to 5-FU treatment. Similar to endogenous *CDC20* promoter, the activity of the luciferase reporter was down-regulated after 5-FU treatment (Figure S9). In two cell types (293, M14) tested, recurrent *CDC20* promoter mutations (including: G25A, G28A, G29A, and GG28/29AA) lead to significantly elevated promoter activity (Figures4A and 4B). However, randomly selected mutation around the consensus sites did not influence luciferase activity (Figure S10). In patient samples with mRNA expression data available (6 samples with *CDC20* promoter hotspot mutation, 27 samples without hotspot mutation), *CDC20* mRNA tend to be up-regulated in melanoma samples with the promoter hotspot mutation (Figure S11) and the difference does not reach statistical significance (unpaired Student’s t-test, p = 0.25), probably due to the limited sample size. Electrophoretic mobility shift assays (EMSA) were performed to analyze changes in protein binding between wild-type and mutant promoters. Results indicate that all tested recurrent *CDC20* promoter mutations have compromised binding affinity to protein factors (Figure 4C).

The four recurrent mutation hotspots in *CDC20* promoter may constitute a single functional protein binding DNA site. Most of the *CDC20* promoter hotspot mutations are identified in patients with melanoma. The prognosis of patients with melanoma with the mentioned *CDC20* promoter hotspot mutations was poorer compared with that of patients without these mutations (Figures 4D and S12); the differences do not reach statistical significance probably due to limited sample size. This implies a function of these *CDC20* promoter mutation hotspots in cancer progression.

### ELK4 binds to the unmutated sequence and represses *CDC20* transcription

The hotspot mutations in *CDC20* promoter are located in the DNA motif GGAAGG, which is predicted to be the binding site for the E26 transformation-specific (ETS) family transcription factors. Mutations in this motif consequently disrupt the binding of ETS transcription factors. To date, 28 ETS transcription factors have been reported in humans (Sizemore et al., 2017). We screened for the potential protein factors that bind to the *CDC20* promoter hotspot mutation-targeted DNA motif based on the following three criteria: (1) the binding sites of the potential transcription factors contain GGAAGG, (2) the potential transcription factors function as transcription repressors, and (3) the potential transcription factors are expressed in melanoma samples. In 28 ETS transcription factors, only six (ERF, ETV3, ELK1, ELK4, ELK3, ETV6) meet the above-mentioned three criteria. Then we experimentally tested the function of these six transcription factors in *CDC20* transcriptional regulation.

**Figure 5:**
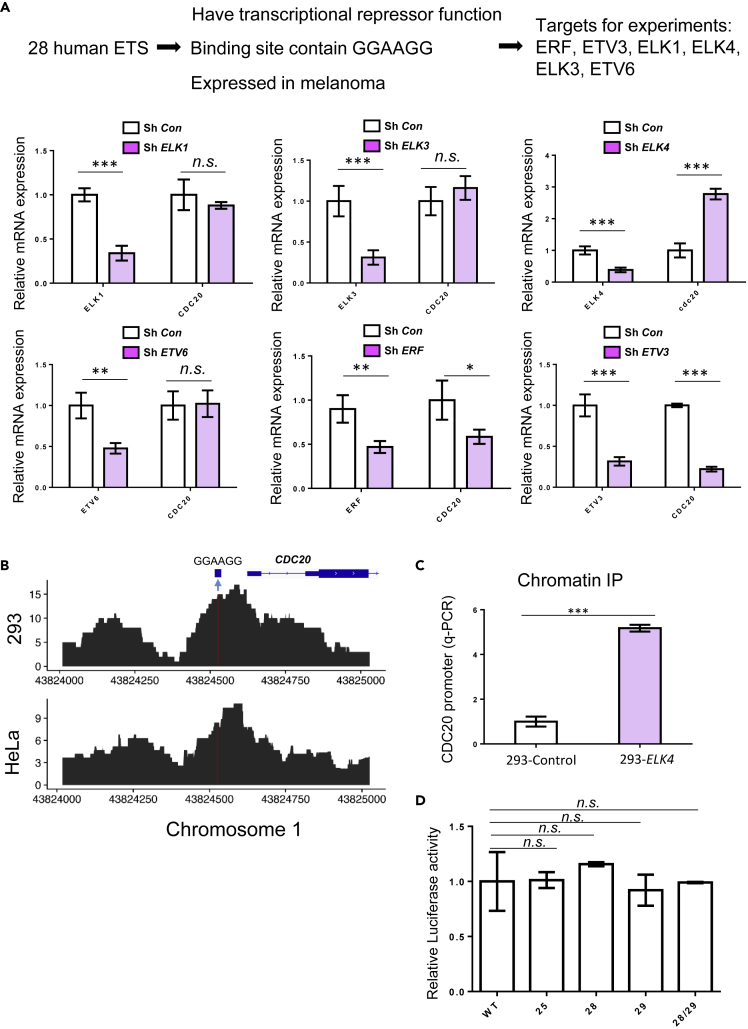
ELK4 binds to the hotspot mutation targeted sequence and represses *CDC20* transcription (A) Screen for ETS proteins that bind the hotspot mutation targeted sequence “GGAAGG” and repress *CDC20* transcription. shRNA experiments were performed in 293 cells; expression of each ETS and *CDC20* mRNA was quantified by qPCR. ∗p < 0.05, ∗∗p < 0.01, ∗∗∗p < 0.001, Student's t test compared to sh-control. The results are an average of three independent experiments. Values are mean ± SD. (B) ENCODE ELK4 ChIP-seq data around the hotspot mutation target sequence “GGAAGG” in 293 and HeLa cells. (C) ChIP was performed with anti-FLAG antibody in M14 cells stably expressing FLAG-ELK4 or FLAG control. The DNA sequence around the hotspot mutation target sequence was quantified with qPCR. ∗∗∗p < 0.001, Student's t test compared with FLAG control. The results are an average of three independent experiments. Values are mean ± SD. (D) Luciferase reporter assay was performed in *ELK4* knockdown 293 cells with wild-type or mutant *CDC20* promoter driving luciferase vectors. The results are an average of three independent experiments. Values are mean ± SD.

We designed short hairpin RNA (shRNA) to knock down the expression of each of the six transcription factors, then checked the expression of *CDC20*, and observed that only knockdown of *ELK4* but not the other five transcription factors resulted in significant up-regulation of *CDC20* transcription (Figure 5A). These data suggest that ELK4 could be the transcription factor that binds to the hotspot mutation targeted motif and suppresses *CDC20* transcription. Based on public ENCODE chromatin immunoprecipitation sequencing (ChIP-seq) datasets, ELK4 binds *CDC20* promoter DNA sequence, and the mutation hotspots are located close to the peak of ELK4 ChIP-seq signals (Figures 5B and S13). The binding between ELK4 and *CDC20* promoter DNA sequence has been experimentally validated with ChIP in M14 cell line (Figure 5C).

In several different cell lines, overexpression of *ELK4* leads to the down-regulation of *CDC20* transcription and knockdown of *ELK4* results in the up-regulation of *CDC20* transcription (Figures S14 and S15). Furthermore, knockdown of *ELK4* can diminish the effects of hotspot mutations on *CDC20* transcription (Figure 5D). These experimental evidences suggest that ELK4 can be the transcription factor that binds the hotspot mutations targeted sequence and suppresses the transcription of *CDC20*.

### Hotspot mutation targeted sequence mediates DNA damage-induced *CDC20* transcription repression

*CDC20* forms a complex with APC/C, and plays a key role in cell cycle spindle checkpoint and metaphase-to-anaphase transition. One of the key physiological functions of APC/C-CDC20 complex is to check the integrity of genome, and DNA damage signal has been reported to dramatically suppress the transcription of *CDC20* (Banerjee et al., 2009). However, the detailed molecular mechanism for this DNA damage-induced *CDC20* transcriptional repression is not clearly understood.

**Figure 6:**
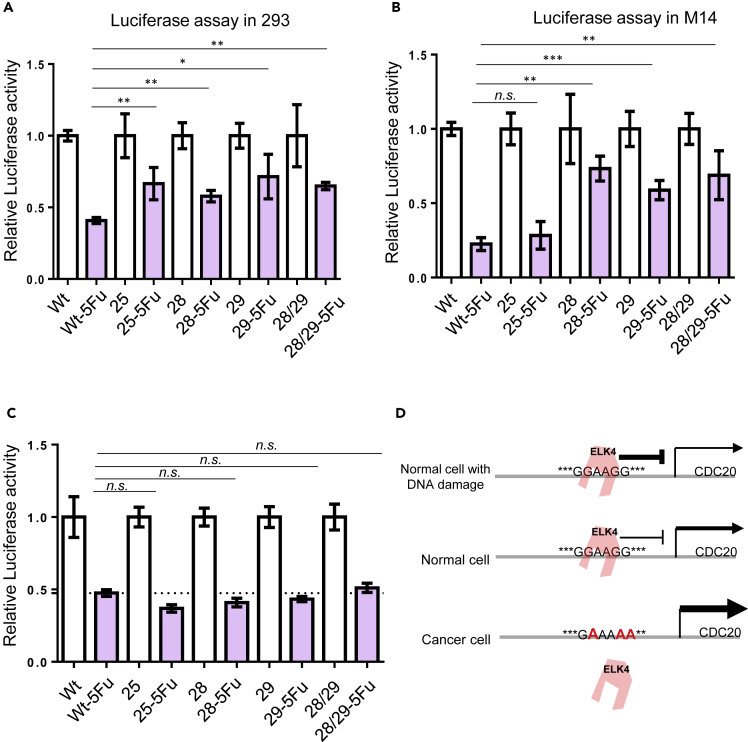
Hotspot mutation targeted sequence mediates DNA damage-induced *CDC20* transcriptional repression (A and B) Luciferase reporter assay was performed in 293 (A) or M14 (B) cells with wild-type or mutant *CDC20* promoter driving luciferase vectors in the presence or absence of DNA damage drug 5-FU. ∗p < 0.05, ∗∗p < 0.01, ∗∗∗p < 0.001, Student's t test compared with wild-type. The results are an average of three independent experiments. Values are mean ± SD. (C) Luciferase reporter assay was performed in *ELK4* shRNA knockdown 293 cells with wild-type or mutant *CDC20* promoter driving luciferase vectors in the presence or absence of 5-FU. (D) Proposed function for the hotspot mutation targeted sequence in *CDC20* transcriptional regulation.

We investigated the consequence of the hotspot mutations on DNA damage-induced *CDC20* transcriptional repression. Using a luciferase reporter assay, the hotspot mutations significantly compromised the effect of DNA damage drug 5-FU on *CDC20* transcriptional suppression (Figures 6A and 6B). This suggested a function of these hotspot mutation targeted sequences in DNA damage-induced *CDC20* transcriptional suppression. *ELK4* knockdown with shRNA also diminishes the effects of hotspot mutations on DNA damage-regulated *CDC20* transcriptional repression (Figure 6C). These experimental evidences suggested that the physiological function of ELK4 binding to the hotspot mutation targeted sequence could be DNA damage-induced *CDC20* transcriptional repression (Figure 6D).

## Discussion

To identify potentially cancer-driving noncoding mutations, we performed pan-cancer WGS analysis with 4,859 samples, the largest number of WGS samples included thus far. We validated known recurrent noncoding mutations. In addition, we identified novel noncoding mutation hotspots, including *CDC20* promoter mutation hotspots, which have been further studied by experiments.

Several recent pan-cancer noncoding studies suggested that cancer noncoding drivers are rare compared with coding drivers (Fredriksson et al., 2014; Rheinbay et al., 2020). One reason might be that these methods did not consider the distinct mutation pattern of noncoding drivers due to the distinct function of noncoding DNA. Many noncoding DNAs act as *cis*-acting element and function by binding protein factors. Our noncoding analysis framework focused on this protein binding function of noncoding DNA. In addition to binding protein factors, noncoding DNA can have a variety of other functions. Some noncoding sequences could have structural function in nucleus organization. For this type of noncoding mutation, we need to focus on the structural effects of genetic alterations. For example, noncoding DNA with a long linear distance can form functional units through 3D interactions, and this type of noncoding driver cannot be identified through conventional linear-based significance analysis. Overall, cancer-driving noncoding mutations may have a different mutation pattern due to different functions. Distinct methods should be applied for identifying those noncoding DNA alterations with distinct functional impacts. However, current methods of cancer noncoding driver discovery did not consider these structural and other functional impacts of noncoding DNA alteration, so it is very likely that many functional noncoding cancer drivers still remain to be discovered.

*CDC20* is a well-known key player in cell cycle regulation. Its expression is frequently up-regulated in various human cancers (Chang et al., 2012; Gayyed et al., 2016; Kim et al., 2014; Wang et al., 2013). Overexpression of *CDC20* is correlated with clinicopathological parameters of various cancers (Wang et al., 2013). *CDC20* inhibitors are in development for the treatment of human cancers (Jiang et al., 2012; Zeng et al., 2010). Importantly, anti-mitotic agents including taxol and nocodazole, which have long been utilized as anticancer reagents, could function by inhibiting APC/C-CDC20 (Huang et al., 2009).

Most of the hotspot mutations in *CDC20* promoter are identified in melanoma samples. Melanoma genomes are known to have high mutation load compared with other cancer types and a predominant C> T nucleotide transition signature attributable to UV radiation (Alexandrov et al., 2013). ETS binding sites in promoter regions are vulnerable to UV mutagenesis (Fredriksson et al., 2017). It is highly possible that these *CDC20* promoter hotspot mutations and other hotspot mutations in melanoma are generated by UV; however, this does not exclude the possibility that some hotspot mutations in transcription factor binding sites can still be functional in cancer evolution, and these need to be tested by experiments. Here we experimentally demonstrated that the *CDC20* promoter hotspot mutations disrupt the binding of transcriptional repressor ELK4, and consequently up-regulate the transcription of *CDC20*. *CDC20* is known to have cancer-driving function through the regulation of cell cycle progression, and consistently *CDC20* expression is ubiquitously up-regulated in various cancer types (Chang et al., 2012; Gayyed et al., 2016; Kim et al., 2014; Wang et al., 2013) (Figure 3A). Thus, the promoter hotspot mutations reported here can promote cancer progression by up-regulating the transcription of *CDC20*.

*CDC20* forms a complex with APC/C and plays a key role in cell cycle spindle checkpoint and metaphase-to-anaphase transition. DNA damage signal has been reported to dramatically suppress the transcription of *CDC20* (Banerjee et al., 2009), and the molecular mechanism for this DNA damage-mediated *CDC20* transcription repression is not clearly understood. The hotspot mutation targeted site reported in this study can mediate the transcriptional repression of *CDC20* induced by DNA damage, and this could be one of the physiological functions of this hotspot mutation targeted DNA site.

Here a noncoding driving mutation analysis framework was developed, which focused on clustered noncoding mutations with potential functional consequence in protein factor binding. This analysis method has implications for the further development of function-based noncoding driver identification pipelines. In addition, recurrent noncoding mutation hotspots were identified in *CDC20* gene promoter; these mutations lead to increased transcription of *CDC20*, which is known to be up-regulated in various cancers and might directly stimulate cancer progression.

### Limitations of the study

The functions of the identified noncoding mutations are evaluated through luciferase reporter assay in this study. The physiological function of these noncoding mutations need to be validated using additional methods, such as generating mutation knockin cell line or knockin animal model. Our *in vitro* experiments suggest that *CDC20* promoter hotspot mutations stimulate *CDC20* transcription, whereas in available human cancer samples with gene expression data, the *CDC20* expression difference between promoter mutated and unmutated samples does not reach statistical significance and more samples are required to fully demonstrate the physiological function of these *CDC20* promoter hotspot mutations in human cancer.

### Resource availability

#### Lead contact

Further information and requests for resources and reagents should be directed to and will be fulfilled by the lead contact, Dr. Xue-Song Liu (liuxs@shanghaitech.edu.cn).

#### Materials availability

All unique reagents generated in this study are available from the lead contact without restriction.

#### Data and code availability

All mutation data used in this analysis were downloaded from ICGC data portal (https://dcc.icgc.org/). Conservation status data can be downloaded from http://hgdownload.soe.ucsc.edu/goldenPath/hg19/phastCons100way/hg19.100way.phastCons.bw. Replication timing data can be downloaded from: http://genome.ucsc.edu/cgi-bin/hgTrackUi?hgsid=686007785_bZhX09eqxKrp5MaaX8giOIZEMx14&c=chr8&g=wgEncodeUwRepliSeq. Mappability data can be downloaded from http://hgdownload.soe.ucsc.edu/goldenPath/hg19/encodeDCC/wgEncodeMapability/wgEncodeCrgMapabilityAlign24mer.bigWig. GC content data can be downloaded from http://hgdownload.soe.ucsc.edu/goldenPath/hg19/gc5Base/hg19.gc5Base.txt.gz. TFBS data can be downloaded from http://hgdownload.cse.ucsc.edu/goldenPath/hg19/encodeDCC/wgEncodeRegTfbsClustered/wgEncodeRegTfbsClusteredWithCellsV3.bed.gz. Data for epigenetic features can be downloaded from https://egg2.wustl.edu/roadmap/data/byFileType/peaks/consolidated/broadPeak/. 1000 Genomes Project phase I data can be downloaded from http://www.internationalgenome.org/data/. All the codes used to reproduce analysis results are freely available at https://github.com/XSLiuLab/Noncoding-code-2020.

## Methods

All methods can be found in the accompanying transparent methods supplemental file.

## Supplemental information

Document S1. Transparent methods and Figures S1–S15

Table S1. List of the clinicopathological parameters of samples with the CDC20 promoter mutations, related to Figure 2
