# Supplementary material for: Pan-cancer noncoding genomic analysis identifies functional CDC20 promoter mutation hotspots
Source: iScience. 2021 Mar 9;24(4):102285. doi: 10.1016/j.isci.2021.102285 (PMC8024666; doi:10.1016/j.isci.2021.102285)
Supplement: Document S1. Transparent methods and Figures S1–S15 [file mmc1.pdf]

iScience, Volume *24*

Supplemental information

Pan-cancer noncoding genomic analysis identifies functional *CDC20* promoter mutation hotspots

Zaoke He, Tao Wu, Shixiang Wang, Jing Zhang, Xiaoqin Sun, Ziyu Tao, Xiangyu Zhao, Huimin Li, Kai Wu, and Xue-Song Liu

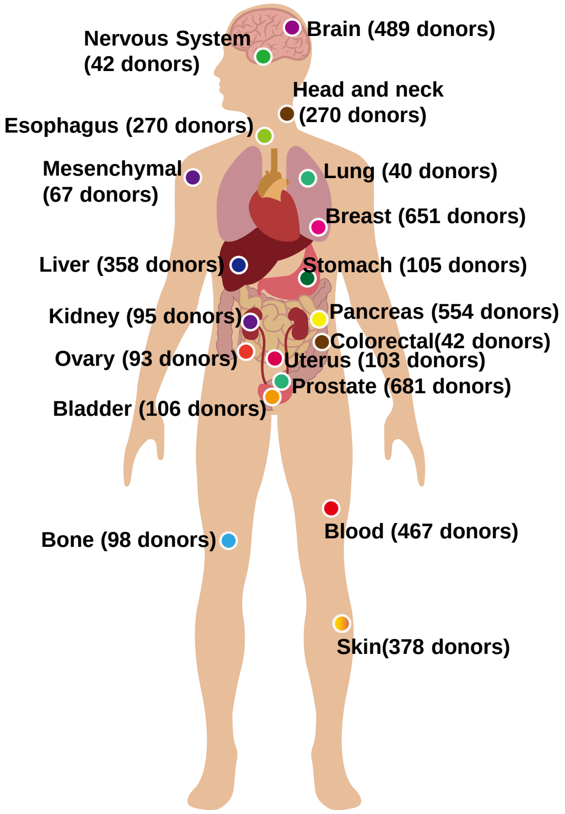

Figure S1. Summary of pan-cancer noncoding analysis data, Related to Figure 1. Number of tumor samples by disease types.

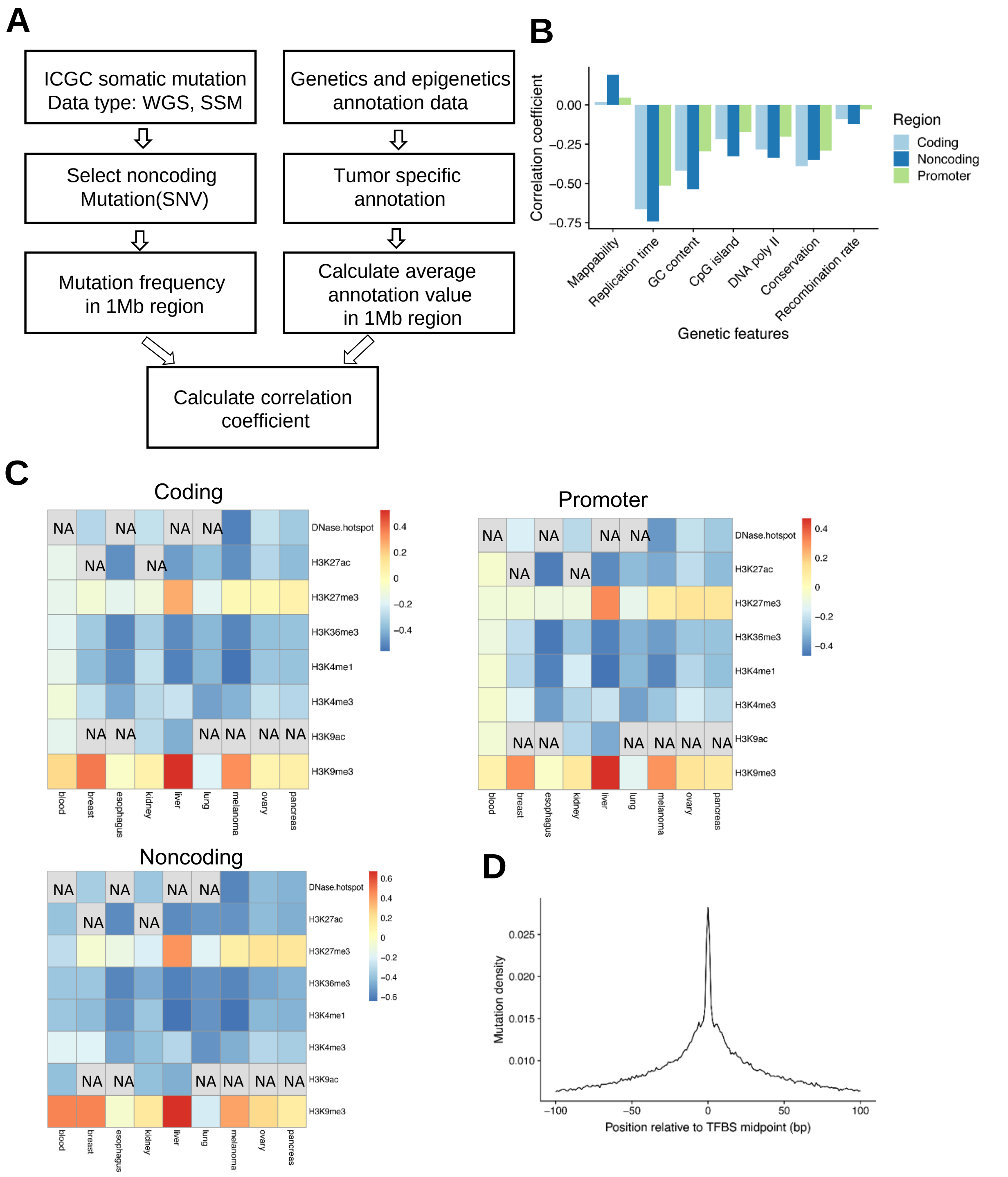

Figure S2. Correlations between coding, noncoding mutation rates and genetic or epigenetic features, Related to Figure 1. (A) Workflow for the correlation analysis between background mutation rates and genetic or epigenetic features. (B) Correlations between genetic features and coding, noncoding, promoter mutation rates. (C) Pearson correlations between epigenetic features and mutation rates in coding, noncoding and promoter regions. (D) Mutation density surrounding TFBS from 4856 ICGC cancer samples.

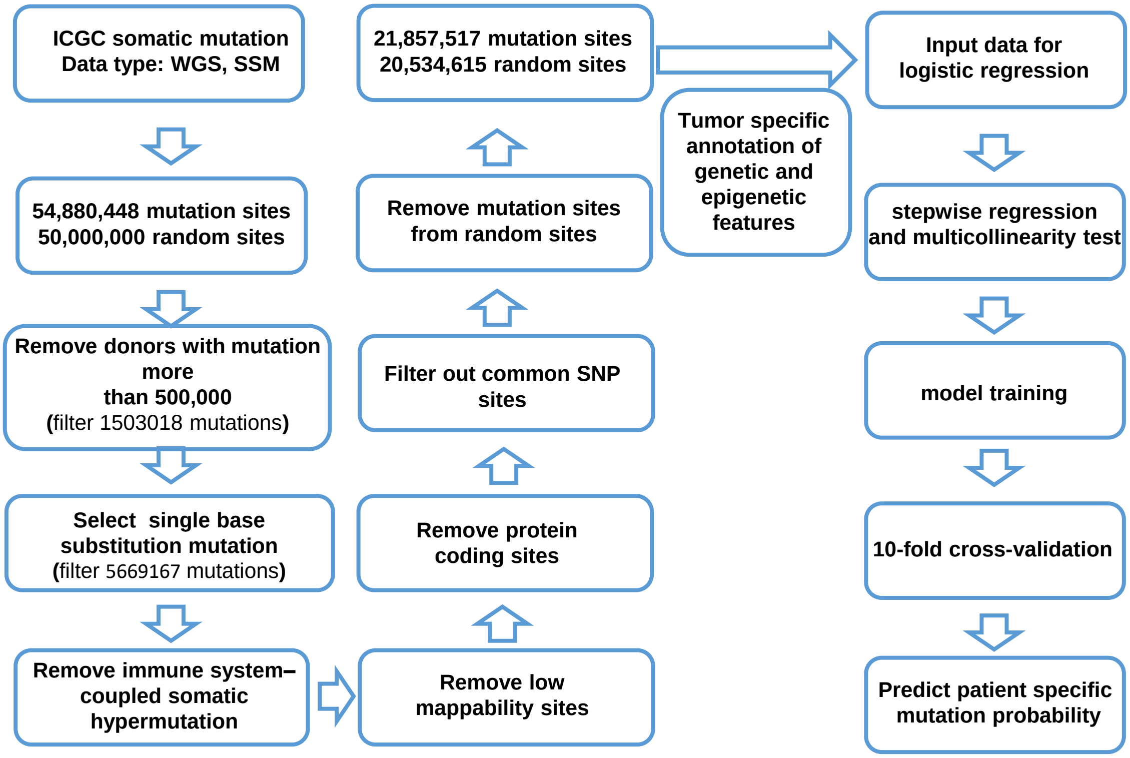

Figure S3. Workflow for the calculation of patient-specific background mutation probability, Related to Figure 1. Flowchart of procedures for calculating patient-specific background mutation probability for noncoding sites using logistic regression model incorporating genetic and epigenetic features of each noncoding site.

Mutation Cluster Region	Nearest Gene	Frequency	P value	Adjust p value
chr5:1295223-1295233	TERT	55	1.93179E-14	6.04843E-10
chr3:16306499-16306510	OXNAD1	39	2.20934E-14	6.91746E-10
chr3:16306499-16306510	DPH3	39	2.20934E-14	6.91746E-10
chr5:1295245-1295255	TERT	39	3.67484E-14	1.15059E-09
chr3:101280665-101280676	TRMT10C	34	1.58762E-14	4.97083E-10
chr8:56987136-56987146	RPS20	27	7.99361E-15	2.5028E-10
chr1:43824520-43824534	CDC20	27	1.4877E-14	4.65799E-10
chr11:47448140-47448154	PSMC3	27	3.66374E-14	1.14712E-09
chr19:17970677-17970687	RPL18A	25	2.27596E-14	7.12602E-10
chr13:41345341-41345351	MRPS31	23	5.55112E-15	1.73805E-10
chr1:155904245-155904255	KIAA0907	23	1.05471E-14	3.3023E-10
chr10:105156311-105156322	PDCD11	22	5.88418E-15	1.84234E-10
chr10:105156311-105156322	USMG5	22	5.88418E-15	1.84234E-10
chr2:32390899-32390910	SLC30A6	21	1.74305E-14	5.45749E-10
chr1:179846979-179846990	TOR1AIP1	20	1.0103E-14	3.16326E-10
chr1:179846979-179846990	TOR1AIP2	20	1.0103E-14	3.16326E-10
chr1:100598548-100598558	TRMT13	20	1.08802E-14	3.40659E-10
chr1:100598548-100598558	SASS6	20	1.08802E-14	3.40659E-10
chr19:7459935-7459946	ARHGEF18	20	1.54321E-14	4.83179E-10
chr9:131038408-131038419	GOLGA2	20	1.82077E-14	5.70082E-10
chr2:70056746-70056757	GMCL1	19	3.44169E-15	1.07759E-10
chr9:35658036-35658047	CCDC107	19	2.14273E-14	6.70889E-10
chr11:98886777-98886796	CNTN5	18	6.99441E-15	2.18995E-10
chr2:26101483-26101494	ASXL2	18	1.25455E-14	3.928E-10
chr22:44208288-44208298	EFCAB6	17	1.24345E-14	3.89324E-10
chr2:176991924-176991943	HOXD8	17	1.25455E-14	3.928E-10
chr12:54582884-54582895	SMUG1	17	1.76525E-14	5.52701E-10
chr19:17970555-17970565	RPL18A	16	6.43929E-15	2.01614E-10
chr15:90931378-90931388	IQGAP1	16	6.66134E-15	2.08566E-10
chr8:114450090-114450103	CSMD3	16	1.12133E-14	3.51087E-10
chr17:7338578-7338588	TMEM102	16	1.31006E-14	4.10181E-10
chr17:7338578-7338588	FGF11	16	1.31006E-14	4.10181E-10
chr6:149867280-149867291	PPIL4	15	6.77236E-15	2.12043E-10
chr12:498771-498781	KDM5A	15	8.77076E-15	2.74613E-10
chr22:31556116-31556126	RNF185	15	8.77076E-15	2.74613E-10
chr1:153963222-153963232	RPS27	15	1.05471E-14	3.3023E-10
chr1:153963222-153963232	RAB13	15	1.05471E-14	3.3023E-10
chr16:27561360-27561371	KIAA0556	14	5.44009E-15	1.70329E-10
chr16:27561360-27561371	GTF3C1	14	5.44009E-15	1.70329E-10
chr1:25559058-25559069	SYF2	14	5.77316E-15	1.80758E-10
chr13:60738136-60738147	DIAPH3	14	8.10463E-15	2.53756E-10
chr22:35795970-35795981	MCM5	14	8.32667E-15	2.60708E-10
chr2:198318139-198318149	COQ10B	14	8.88178E-15	2.78089E-10
chr16:67260975-67260988	TMEM208	14	1.14353E-14	3.58039E-10
chr16:67260975-67260988	AC040160.1	14	1.14353E-14	3.58039E-10
chr16:67260975-67260988	LRRC29	14	1.14353E-14	3.58039E-10
chr17:1588271-1588281	PRPF8	14	1.37668E-14	4.31037E-10
chr11:73309651-73309662	FAM168A	13	6.32827E-15	1.98138E-10
chr10:18940596-18940606	NSUN6	13	6.88338E-15	2.15519E-10

Figure S4. List of significantly mutated noncoding regions, Related to Figure 2. Eleven base pair Noncoding DNA regions are first ranked based on mutation probability, and top 50 (-Log_{10} (P Value)) noncoding regions are further ranked based on mutation frequency. Nearest genes to each noncoding regions are also shown.

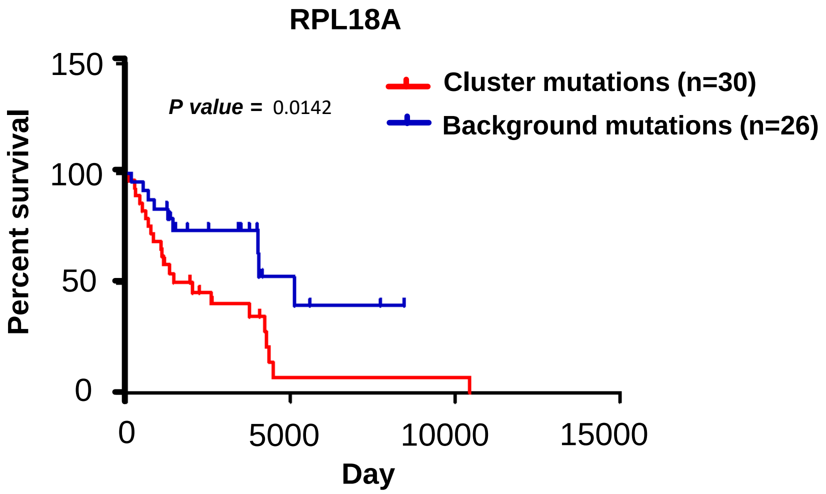

Figure S5. Kaplan–Meier overall survival curves of melanoma patients with indicated *RPL18A* promoter mutations or other mutations in the background, Related to Figure 2. n=30 for melanoma patients with clustered *RPL18A* promoter mutations (Chr19: C17970682T and G17970560A) and n=26 for melanoma patients with other mutations in the background region. Log-rank (Mantel-Cox) test *P* value is shown.

7bp window

Mutation Cluster Region	gene_name	count	adj_p_val
chr5:1295225-1295231	TERT	55	3.13E-10
chr3:16306501-16306508	OXNAD1	39	5.15E-10
chr3:16306501-16306508	DPH3	39	5.15E-10
chr5:1295247-1295253	TERT	38	3.06E-10
chr3:101280667-101280674	TRMT10C	34	7E-10
chr11:47448142-47448152	PSMC3	27	7.94E-10
chr1:43824522-43824532	CDC20	26	1.74E-10
chr8:56987138-56987144	RPS20	26	1.21E-09
chr19:17970679-17970685	RPL18A	25	7.56E-10
chr13:41345343-41345349	MRPS31	23	7E-304
chr1:155904247-155904253	KIAA0907	23	1.03E-09
chr10:105156313-105156320	PDCD11	22	1.02E-09
chr10:105156313-105156320	USMG5	22	1.02E-09
chr2:32390901-32390908	SLC30A6	20	1.21E-09
chr1:100598550-100598556	TRMT13	19	5.36E-10

9bp window

Mutation Cluster Region	gene_name	count	adj_p_val
chr5:1295224-1295232	TERT	55	6.26E-11
chr5:1295246-1295254	TERT	39	4.38E-10
chr3:16306500-16306509	OXNAD1	39	6.89E-10
chr3:16306500-16306509	DPH3	39	6.89E-10
chr3:101280666-101280675	TRMT10C	34	8.21E-10
chr11:47448141-47448153	PSMC3	27	1.12E-09
chr1:43824521-43824533	CDC20	26	4.87E-10
chr8:56987137-56987145	RPS20	26	1.16E-09
chr19:17970678-17970686	RPL18A	25	6.99E-10
chr13:41345342-41345350	MRPS31	23	1.04E-11
chr1:155904246-155904254	KIAA0907	23	9.98E-10
chr10:105156312-105156321	PDCD11	22	8.73E-10
chr10:105156312-105156321	USMG5	22	8.73E-10
chr2:32390900-32390909	SLC30A6	21	1.12E-09
chr1:100598549-100598557	TRMT13	20	4.7E-10

13bp window

Mutation Cluster Region	gene_name	count	adj_p_val
chr5:1295222-1295234	TERT	55	2.81E-10
chr5:1295244-1295256	TERT	39	3.58E-10
chr3:16306498-16306511	OXNAD1	39	8.09E-10
chr3:16306498-16306511	DPH3	39	8.09E-10
chr3:101280664-101280677	TRMT10C	34	8.89E-10
chr1:43824519-43824535	CDC20	27	5.97E-10
chr11:47448139-47448155	PSMC3	27	9.31E-10
chr8:56987135-56987147	RPS20	27	1.11E-09
chr19:17970676-17970688	RPL18A	25	1.07E-09
chr13:41345340-41345352	MRPS31	23	1.04E-11
chr1:155904244-155904256	KIAA0907	23	1.09E-09
chr10:105156310-105156323	PDCD11	22	8.54E-10
chr10:105156310-105156323	USMG5	22	8.54E-10
chr2:32390898-32390911	SLC30A6	21	1.06E-09
chr9:35658035-35658048	CCDC107	20	2.95E-10

15bp window

Mutation Cluster Region	gene_name	count	adj_p_val
chr5:1295221-1295235	TERT	55	2.67E-10
chr5:1295243-1295257	TERT	39	5.76E-10
chr3:16306497-16306512	OXNAD1	39	7.85E-10
chr3:16306497-16306512	DPH3	39	7.85E-10
chr3:101280663-101280678	TRMT10C	34	8.65E-10
chr8:56987134-56987148	RPS20	30	1.14E-09
chr1:43824518-43824536	CDC20	27	4.13E-10
chr11:47448138-47448156	PSMC3	27	1.01E-09
chr19:17970675-17970689	RPL18A	25	9.06E-10
chr13:41345339-41345353	MRPS31	23	7E-304
chr1:155904243-155904257	KIAA0907	23	7.85E-10
chr10:105156309-105156324	PDCD11	22	1.03E-09
chr10:105156309-105156324	USMG5	22	1.03E-09
chr11:98886775-98886798	CNTN5	21	6.77E-10
chr2:32390897-32390912	SLC30A6	21	9.41E-10

17bp window

Mutation Cluster Region	gene_name	count	adj_p_val
chr5:1295220-1295236	TERT	55	2.67E-10
chr5:1295242-1295258	TERT	39	5E-10
chr3:16306496-16306513	OXNAD1	39	7.98E-10
chr3:16306496-16306513	DPH3	39	7.98E-10
chr3:101280662-101280679	TRMT10C	34	9.99E-10
chr8:56987133-56987149	RPS20	30	1.15E-09
chr1:43824517-43824537	CDC20	28	3.75E-10
chr11:47448137-47448157	PSMC3	27	9.86E-10
chr19:17970674-17970690	RPL18A	25	1.13E-09
chr1:155904242-155904258	KIAA0907	24	1.01E-09
chr13:41345338-41345354	MRPS31	23	3.82E-11
chr9:35658033-35658050	CCDC107	22	5.14E-10
chr10:105156308-105156325	PDCD11	22	1.09E-09
chr10:105156308-105156325	USMG5	22	1.09E-09
chr11:98886774-98886799	CNTN5	21	4.65E-10

21bp window

Mutation Cluster Region	gene_name	count	adj_p_val
chr5:1295218-1295238	TERT	55	4.44E-10
chr5:1295240-1295260	TERT	39	7.49E-10
chr3:16306494-16306515	OXNAD1	39	9.01E-10
chr3:16306494-16306515	DPH3	39	9.01E-10
chr3:101280660-101280681	TRMT10C	34	9.74E-10
chr8:56987131-56987151	RPS20	30	1.06E-09
chr1:43824515-43824539	CDC20	28	6.07E-10
chr16:67260952-67261010	TMEM208	27	8.11E-10
chr16:67260952-67261010	AC040160.1	27	8.11E-10
chr16:67260952-67261010	LRRC29	27	8.11E-10
chr1:153963184-153963237	RPS27	27	9.15E-10
chr1:153963184-153963237	RAB13	27	9.15E-10
chr11:47448135-47448159	PSMC3	27	1.17E-09
chr19:17970672-17970692	RPL18A	25	9.5E-10
chr1:155904240-155904260	KIAA0907	24	9.46E-10

Figure S6. List of significantly mutated noncoding regions calculated with different size of window (From 7bp to 21bp window), Related to Figure 2. Noncoding DNA regions are first ranked based on mutation probability, and top 50 ($-Log_{10}$ (P Value)) noncoding regions are further ranked based on mutation frequency.

Regions	gene_name	count	p_val	adj_p_val
chr3:46780065-46780075	PRSS46	9	1.9873E-14	1.3911E-11
chr3:167375318-167375328	WDR49	6	1.5654E-14	1.0958E-11
chr8:42399654-42399664	SLC20A2	6	2.7756E-14	1.9429E-11
chr3:11765471-11765481	VGLL4	5	1.5654E-14	1.0958E-11
chr1:156859617-156859627	PEAR1	5	2.0095E-14	1.4067E-11
chr3:11034286-11034296	SLC6A1	5	2.6312E-14	1.8419E-11
chr14:21078826-21078836	RNASE11	5	4.4368E-12	3.1057E-09
chr14:21078826-21078836	RNASE11	5	4.4368E-12	3.1057E-09
chr12:10162489-10162499	CLEC12B	5	1.1796E-09	8.2569E-07
chr11:48388090-48388100	OR4C5	4	3.0198E-14	2.1139E-11
chr13:106115297-106115307	DAOA	4	6.2506E-14	4.3754E-11
chr11:118174980-118174990	CD3E	4	5.258E-13	3.6806E-10
chr20:63544-63554	DEFB125	4	7.9448E-13	5.5613E-10
chr19:52040086-52040096	SIGLEC6	4	1.5451E-12	1.0816E-09
chr19:48763863-48763873	CARD8	4	3.4791E-12	2.4354E-09
chr10:124765924-124765934	ACADSB	4	1.3451E-11	9.4155E-09

Figure S7. List of significantly mutated noncoding indels calculated with 11bp window, Related to Figure 2. Noncoding DNA regions with clustered indels in 11bp window are first ranked based on indel probability, and top 50 (-Log_{10} (P Value)) noncoding regions are further ranked based on the frequency of indel.

Mutation Cluster Region	gene_name	sequenceType	count	p_val	adj_p_val
chr5:1295223-1295233	TERT	promoter	55	1.07E-14	3.34E-10
chr1:203275149-203275166	BTG2	intron	41	1.14E-14	3.58E-10
chr5:1295245-1295255	TERT	promoter	39	1.12E-14	3.51E-10
chr3:16306499-16306510	OXNAD1	promoter	39	2.64E-14	8.27E-10
chr3:16306499-16306510	DPH3	promoter	39	2.64E-14	8.27E-10
chr19:10340883-10340911	S1PR2	intron	36	1.47E-14	4.59E-10
chr1:203275100-203275113	BTG2	intron	36	2.25E-14	7.06E-10
chr1:203274969-203274988	BTG2	intron	34	1.09E-14	3.41E-10
chr3:101280665-101280676	TRMT10C	promoter	34	2.96E-14	9.28E-10
chr1:203275555-203275578	BTG2	intron	33	2.08E-14	6.5E-10
chr9:37026307-37026318	PAX5	intron	28	1.45E-14	4.55E-10
chr2:136875308-136875336	CXCR4	intron	27	1.17E-14	3.65E-10
chr1:43824520-43824534	CDC20	promoter	27	1.2E-14	3.75E-10
chr11:47448140-47448154	PSMC3	promoter	27	3.66E-14	1.15E-09
chr8:56987136-56987146	RPS20	5_utr	27	3.86E-14	1.21E-09
chr8:56987136-56987146	RPS20	promoter	27	3.86E-14	1.21E-09
chr1:240636863-240636873	FMN2	intron	26	8.66E-15	2.71E-10
chr16:10973681-10973696	CIITA	intron	25	1.51E-14	4.73E-10
chr1:203275173-203275197	BTG2	intron	25	1.89E-14	5.91E-10
chr19:17970677-17970687	RPL18A	promoter	25	2.78E-14	8.69E-10
chr19:17970677-17970687	RPL18A	5_utr	25	2.78E-14	8.69E-10
chr10:115511585-115511598	PLEKHS1	intron	24	8.55E-15	2.68E-10
chr13:41345341-41345351	MRPS31	5_utr	23	0	7E-304
chr13:41345341-41345351	MRPS31	promoter	23	0	7E-304
chr1:999995-1000005	BC033949	intron	23	0	7E-304
chr1:155904245-155904255	KIAA0907	promoter	23	3.06E-14	9.59E-10
chr5:22578492-22578502	CDH12	intron	23	3.18E-14	9.94E-10
chr1:203275075-203275090	BTG2	intron	22	8.88E-15	2.78E-10
chr6:91005801-91005819	BACH2	intron	22	1.28E-14	4E-10
chr2:136875033-136875051	CXCR4	intron	22	1.78E-14	5.56E-10
chr9:37025356-37025366	PAX5	intron	22	2E-14	6.26E-10
chr1:203274951-203274966	BTG2	intron	22	2.08E-14	6.5E-10
chr6:6307789-6307799	F13A1	intron	22	2.12E-14	6.64E-10
chr2:77611356-77611366	LRRTM4	intron	22	2.24E-14	7.02E-10
chr10:105156311-105156322	PDCD11	promoter	22	2.69E-14	8.41E-10
chr10:105156311-105156322	USMG5	promoter	22	2.69E-14	8.41E-10
chr2:32390899-32390910	SLC30A6	promoter	21	3.15E-14	9.87E-10
chr2:32390899-32390910	SLC30A6	5_utr	21	3.15E-14	9.87E-10
chr7:137139140-137139152	DGKI	intron	21	3.28E-14	1.03E-09
chr1:203275118-203275140	BTG2	intron	21	3.38E-14	1.06E-09
chr6:125531722-125531734	TPD52L1	intron	20	1.14E-14	3.58E-10
chr13:33608075-33608085	KL	intron	20	1.58E-14	4.94E-10
chr9:37026725-37026736	PAX5	intron	20	1.75E-14	5.49E-10
chr8:2031625-2031656	MYOM2	intron	20	2.23E-14	6.99E-10
chr1:100598548-100598558	TRMT13	promoter	20	2.26E-14	7.09E-10
chr1:100598548-100598558	SASS6	promoter	20	2.26E-14	7.09E-10
chr1:179846979-179846990	TOR1AIP1	promoter	20	2.71E-14	8.48E-10
chr1:179846979-179846990	TOR1AIP2	promoter	20	2.71E-14	8.48E-10
chr9:131038408-131038419	GOLGA2	promoter	20	2.76E-14	8.66E-10
chr2:136874959-136874978	CXCR4	intron	20	3.29E-14	1.03E-09

Figure S8. List of significantly mutated noncoding mutations in 5'-UTR, 3'-UTR and intron regions, Related to Figure 2. Noncoding DNA regions are first ranked based on mutation probability in 11bp window, and top 50 (-Log_{10} (P Value)) noncoding regions are further ranked based on mutation frequency.

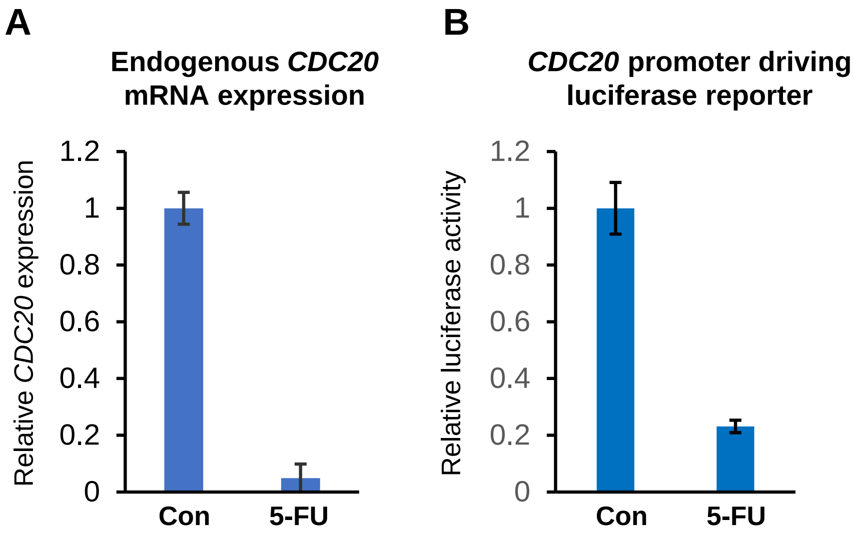

Figure S9. *CDC20* promoter driving luciferase reporter can mimic the response of human endogenous *CDC20* promoter in response to DNA damage drug, Related to Figure 4.

(A) Endogenous *CDC20* mRNA expression in response to DNA damage drug 5-FU in M14 cells. The expressions of *CDC20* mRNA were quantified by Q-PCR.

(B) Luciferase activity of cloned *CDC20* promoter-driving reporter in response to 5-FU in M14 cells. Error bars represent mean ± s.d. from three experiments.

A

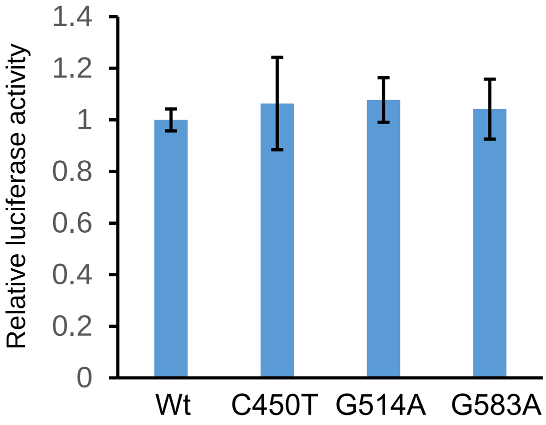

B

ATTCCCACAAACTTCTTTGTATGACCTCGGGCACATCCCTTCCCTGGGCCTCCGTTTCTCCATCTGTAAAAT
ATGGATTTGTTGTCGGGGTGGGGAGGCTGCACCACGCACAGGTTAGACTAATGGATCTCTAAGGTCCTCA
CATCTTTAAAGCCCCAAGGGGATAAGCCACAGTGCCTCCTGTAGGGCAGTCTAAGCTTATCTTCCAGATA
GGCAGGTTTGAATACCGATCCTTTTTCTTGACCTTAAGGAATTCATTCACCCTTTTCAACCTCATTTTCCCTG
TTTGTAAAACAACAGCAAACGAGACAAACACACGTTACTTCCTTTCTAGCAGGGTTCCTACCCGGCGCCAA
GCAAAAGTGGAATGTACCCTAAGTAGCTCTGGCCTTCTTCCTGCTCCCAAGCTTCCCAATTCCGTCCCCTGC
CCCGCTGCCGCCCGCGGCTCTCCTTCCCCTTCTAGGAACGGCTCAAGCGCCTTGGGCACTCCATCGGGTTC
TGCACCGAGTTCTGCATCATAAATACGACTCTCGTGTAGGATTTAAGTGTGAACTTCTGCAGGTTCTCGGA
CCCTGAAGCACCCGGGGCCAGACATTCCGAGCTCGCGGCGGTGGAAGGCACGCAAAAGGGCGAACCGA
GACGACTCCAGGACGCTGAGGCAGCGCAGGCCCCACCCGGCCCCGCCCTGCCCCGCCCTGTCCCGGCCGG
CTTTCCAGTACTAGTCCTCTGGCGC**C**(C450T)GGCTCCCAGCCCCTCTCGTACCCTTCAAATCGCGCTCCG
CCGCTAGACTCTCGTGATAGCTGA**G**(G514A)ACTTTCCCCG**G**AA**GG**CCCGCCCCCTTCGCCGGAGAG
GCCAATGGGCTAGGGCAACGGTTGCGACGGTT**G**(G583A)GATTTTGAAGGAGCCAATAGGCGCTCGG
AGCGGAGAGTTTAAGAGGCGTAAGCCAGGCGTGTTAAAGCCGGTCGGAACTGCTCCGGAGGGCACGGT
GAGAGGTGGTGGGGCTGAGCCGAGGTGGGGCCGTGGCCAGGGGGAGGGGGTGCTAGGCCGGAAGGG
GCTGCAGCCGAGGGTGGCCCTGATTTTGTGGCCGGCCAGGAGCGAAGGGGTCCCTTTCTGTCCCCTGAGC
AC

Figure S10. Random mutations in *CDC20* promoter-driving luciferase reporter did not influence luciferase activity, Related to Figure 4.

(A) Luciferase reporter assay was performed with wild-type promoter or C450T, G514A, G583A mutations in M14 cells. Error bars represent mean ± s.d. from three experiments.

(B) *CDC20* promoter sequence used for luciferase reporter assay is shown, and the locations of each mutations are labeled.

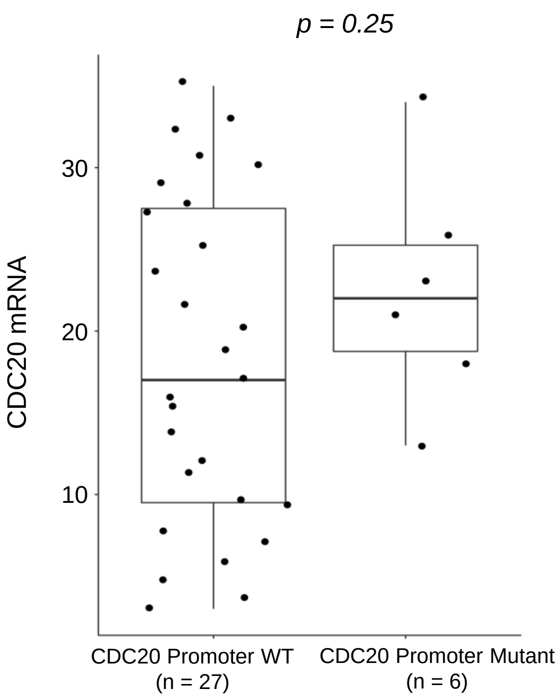

Figure S11. CDC20 mRNA levels in melanoma samples with or without the promoter hotspot mutations, Related to Figure 4. In total 6 samples with the CDC20 promoter hotspot mutations and 27 samples without the promoter hotspot mutations have gene expression data available for analysis. *P* value is calculated with unpaired, two-tailed Student's t test.

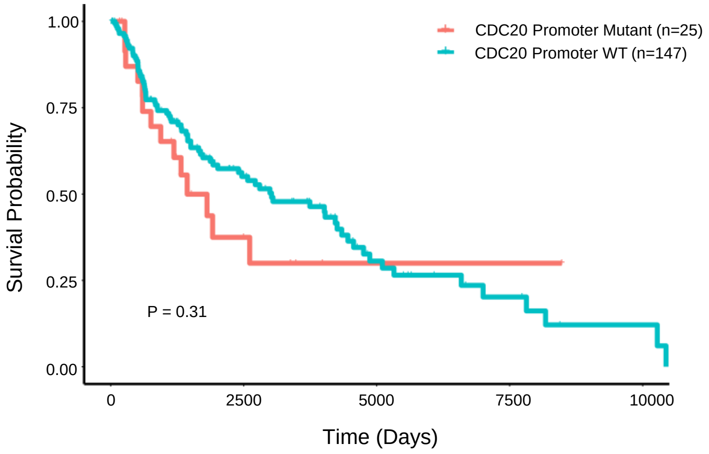

Figure S12. Kaplan–Meier overall survival curves of melanoma patients with indicated *CDC20* promoter mutations or control mutations, Related to Figure 4. n=25 for patients with clustered *CDC20* promoter mutations (including G25A, G28A, G29A and GG28/29AA), n=147 for patients without the clustered promoter mutations. Log-rank (Mantel-Cox) test *P* value is shown.

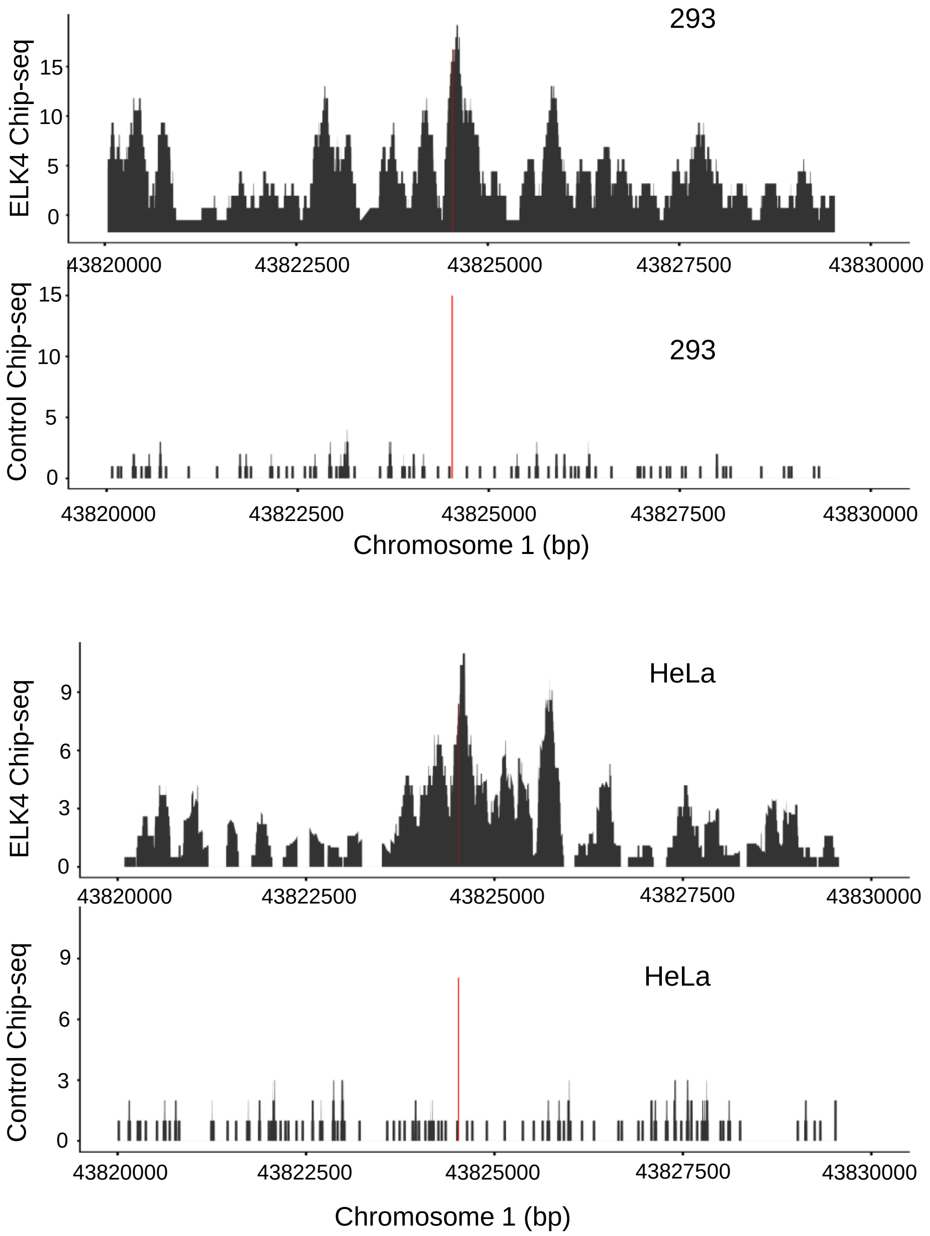

Figure S13. A zoomed out version of Figure 5B is shown, Related to Figure 5. ENCODE ELK4 and control ChIP-seq data around the hotspot mutation target sequence "GGAAGG" (marked as red line) in 293 and HeLa cells.

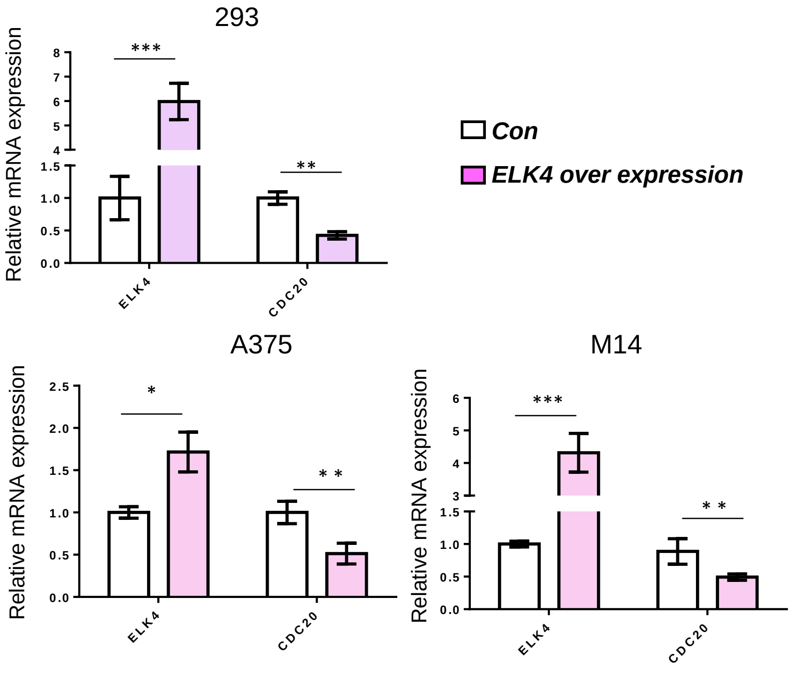

Figure S14. Overexpression of *ELK4* suppresses *CDC20* in multiple cell lines, Related to Figure 5. The expression of *ELK4* and *CDC20* mRNA were quantified by Q-PCR.

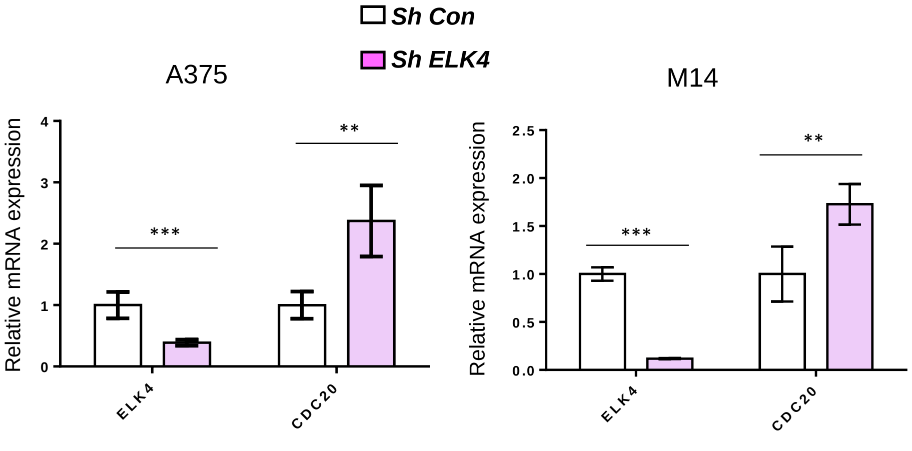

Figure S15. Knockdown of *ELK4* stimulates *CDC20* transcription in cell lines, Related to Figure 5. *ELK4* was knockdown with shRNA, the expression of *ELK4* and *CDC20* mRNA were quantified by Q-PCR.

Transparent Methods

Cancer genome data preprocessing

The reference genome used throughout this study is hg19. We downloaded cancer whole-genome sequencing (WGS) data from International Cancer Genome Consortium (ICGC) release 27. In total, there were 4,881 donors, 54,880,488 mutation sites and 59,699,855 mutations before data preprocessing. Nine samples with more than 500,000 mutations were excluded to eliminate ultra-mutated samples. We extracted mutation type "*single base substitution*" (point mutations) for analysis, and several samples without single base substitution have been removed from analysis. Common human SNP variants were removed from the cancer genome mutation datasets based on 1000 Genomes Project (Genomes Project et al., 2015). We also removed the immunoglobulin loci region according to the Ensembl (v75) annotation from further analysis to avoid bias from immune system-coupled somatic hypermutation. The final mutation data was converted to BED format for subsequent analysis. In total 4859 samples with 47,708,263 mutations are included in downstream analysis.

Genetic and epigenetic features as covariates of background mutation rates

We used a variety of annotation features to analyze background mutation rates. These features can be roughly divided into genetic features and epigenetic features. The values of genetic features are determined by the genomic DNA sequence, and are thus consistent in different tumor types. The values of epigenetic features show variations among cancer types with different tissue origins. The values of these annotation features were downloaded from UCSC genome browser database or ENCODE database, and are described as below.

Sequence context: We used the 3 base pairs nucleotide motifs centered at the mutated site (1-bp left/right flank motifs of the site). Reverse compliment pairs are combined together, in total there are 32 types of sequence contexts.

Genome mappability: This feature refer to the uniqueness of DNA sequence in mapping with reference genome. Genome mappability data was downloaded from UCSC Genome Browser.

Recombination rate: Recombination in meiosis help to expand genetic diversity. In somatic cells, DNA lesions can be repaired through recombination between homologous chromosomes. Recombination rate data was downloaded from UCSC Genome Browser.

Conservation: We used phastCons data (hg19.100way.phastCons.bw)

downloaded from UCSC genome browser to reflect the conservation status of genomic DNA. PhastCons estimates the probability that each nucleotide belongs to a conserved element, based on a phylogenetic hidden Markov model (Siepel et al., 2005).**Error! Reference source not found.**

Replication timing: We used the ENCODE replication timing data downloaded from the UCSC genome browser. The average wavelet-smoothed signals of repli-seq from 14 cell lines: BJ, GM06990, GM12801, GM12812, GM12813, GM12878, HeLa-S3, HepG2, HUVEC, IMR-90, K-562, MCF-7, NHEK and SK-N-SH were used to assess the genome-wide DNA replication timing.

GC contents: We used GC content raw data in UCSC genome browser to calculate GC content. The file hg19.gc5Base.txt.gz contains the GC content for 5bp windows across whole genome was downloaded with hgGcPercent.

CpG islands: We used UCSC Genome Browser tools to download CpG islands data. The selection criteria is “Mammal”, “Human”, “GRCH37/hg19”, “Regulation”, “CpG Islands”.

Promoters: We selected RefSeq-defined human protein coding genes for analysis. Promoter was defined as the region from 2,500 base pair (bp) upstream to 500 bp downstream from the annotated transcript start site. Pseudogenes are known hot spots for artifacts due to their sequence similarity to their parent genes. In order to avoid potential variant calling bias, partially due to mapping difficulty, we removed the promoters and UTR analyses for pseudogenes.

Transcription factor binding sites (TFBS): TFBS information is based on data from ChIP-seq experiments performed by the ENCODE project (Consortium, 2012). ENCODE union TFBS regions processed by FunSeq (http://funseq2.gersteinlab.org/data/2.1.0) were analyzed in this study. The midpoint of each TFBS was determined by averaging the start and end position of the binding site.

DNA polymerase II: We used data from ChIP-seq experiments performed by the ENCODE project. The average value of uniform peak signals for 4 cell lines, K562, MCF10A, PBDE and Raji were used for analysis.

The epigenetic features of the genome were downloaded from Roadmap Epigenomics Project. For pan-cancer analysis, we used the data from integrative analysis of 111 reference human epigenomes (Roadmap Epigenomics et al., 2015). Chromatin accessibility (DNase-seq) and seven types of histone modifications (H3K4me1, H3K4me3, H3K27me3, H3K36me3, H3K9me3, H3K27ac and H3K9ac) data are included in downstream analysis. The epigenome identifier from release 9 of the compendium (Roadmap

Epigenomics et al., 2015) for each tumor types are shown below: breast (E028), esophagus (E079), kidney (E086), liver (E066), lung (E096), melanoma (E059 and E061), ovary (E097), pancreas (E098).

Patient-specific background mutation probability model

We used logistic regression model to estimate the background mutation probability for each genome site. The expected background mutation rates are modeled using genetic and epigenetic features that co-vary with the localized mutation rates. We removed CDS region and immunoglobin loci, and selected high-mappable regions from whole genome for the logistic regression model. Replication timing, genetic features, epigenetic features and patient ID information are included in the logistic regression model to calculate the expected patient-specific mutation rate for each genome site.

Poisson binomial model for mutation significance

We selected all single base substitutions with recurring frequency more than 3 and extended 5-bp left/right flank to get the 11bp regions as candidate clustered mutation regions. Noncoding mutation within 5kb of gene transcription start sites are further selected in downstream analysis. For each 11bp region, we calculated the mutation probability of each genome site using logistic regression model, then calculated the mutation probability for each 11bp region:

$$\Pr(\text{region is mutated}) = 1 - \prod_{i=1}^{11}(1 - p_i)$$

Here p_i is the mutation probability of genome site i within the 11bp region. Mutation recurrence in the given region of interest is then modeled using the Poisson binomial distribution, which accounts for variations in mutation rates across tumors. For a specific region of interest, the probability of having mutations in k or more individuals is calculated as following:

$$\Pr(K \geq k) = \sum_{m=k}^{n} \sum_{A \in F_m} \prod_{i \in A} p_i \prod_{j \in A^c} (1 - p_j)$$

Here, p_i and p_j are the region mutation probabilities for different patients, n is the total number of patients, k is the patient number with mutation in the given 11bp region. We used the R package "poibin" to calculate the *P* value for each 11bp region (Hong, 2013). The *P* values were then adjusted with Bonferroni method.

CDC20 promoter related database analysis

CDC20 mRNA expression analysis in pan-cancer: We used the Firebrowse database of Broad Institute to compare the mRNA expression difference between tumor and normal tissues in 37 cancer types. The mRNA expression levels are represented as normalized RSEM (log_2).

Survival analysis: TCGA SKCM patients were selected and divided into two groups, *CDC20* mRNA high and *CDC20* mRNA low, based on *CDC20* mRNA expression level. Kaplan-Meier overall survival curves were compared in these two groups. Log-rank test *P* value was reported. In ICGC MELA-AU project, we selected patients with mutation occurred in *CDC20* locus and nearby regions. Then we divided the patients into two groups, one group with mutation in *CDC20* promoter mutation hotspot region, another group with mutation occurred in *CDC20* locus but not in promoter mutation hotspot region. Kaplan-Meier overall survival curves were compared in these two groups.

ELK4 Chip-seq signal visualization: Two ELK4 Chip-Seq datasets including HeLa-S3 and HEK293 cell lines were queried by https://www.encodeproject.org/search/?searchTerm=ELK4&type=Dataset, bigWig files were downloaded and the ELK4 signals around CDC20 promoter were then plotted with R.

CDC20 promoter cloning and mutation

CDC20 promoter containing 859 bp upstream of the transcription start site was amplified from human genomic DNA with the primers 5'ATGCGGTACCGGCAGTCTAAGCTTATCTTCCAGATA3' and 5'ATGCCTCGAGGTGCTCAGGGGACAGAAAGGGACC3'. The amplified fragment was cloned into the mammalian expression vector pGL3 basic from Promega using the restriction enzymes KpnI and XhoI. The site directed mutations of *CDC20* promoter were created using the Fast Mutagenesis System from Transgen according to manufacturer`s protocol. The primers used for mutation are listed below:

525 F-5'CTGAGACTTTCCCCGAAAGGCCCGCCR3',
R-5'TCGGGGAAAGTCTCAGCTATCACGA3';
528 F- 5'AGACTTTCCCCGGAAAGCCCGCCCCC3',
R-5'TTTCCGGGGA AAGTCTCAGCTATCA3' ;
529 F-5'GACTTTCCCCGGAAGACCCGCCCCCT3',
R- 5'TCTTCCGGGG AAAGTCTCAGCTATC3'.
450-F: TCCTCTGGCGCTGGCTCCCAGC
R: GCTGGGAGCCAGCGCCAGAGGA

M514-F: TGATAGCTGAAACTTTCCCCGG
R: CCGGGGAAAGTTTCAGCTATCA
M583-F: GCGACGGTTAGATTTTGAAG
R: CTTCAAAATCTAACCGTCGC
All constructed vectors have been validated by sequencing.

Luciferase reporter assay

Five thousand cells (HEK293, M14) per well were co-transfected in 96-well format with wild type or mutant *CDC20* promoter driving pGL3 vector and Renilla plasmid as a normalization control. Forty eight hours after transfection the cells were washed with phosphate-buffered saline (PBS). The cells were then lysed in the luciferase lysis buffer provided with the Luciferase Assay Kit (Promega, Madison, USA). Luciferase activity was measured with the Dual-Luciferase Reporter Assay System (Promega). Values reported are firefly luciferase divided by Renilla luciferase. All cell lines were obtained from ATCC and were cultured in DMEM (Corning, Cellgro) plus 10% FBS (Gibco), 100 U/ml penicillin G and 100 μg/ml streptomycin (Corning, Cellgro). Each assay was done in duplicate and repeated for three times.

Quantitative PCR (Q-PCR) to quantify gene expression

Total RNA was extracted with TRIzol® Reagents (Invitrogen) according to the provided protocol. 1μg total RNA was reversed transcribed with iScript™ cDNA Synthesis Kit (Bio-Rad). Real time quantitative PCR was performed using diluted cDNA, SYBR® Green JumpStart™ Taq ReadyMix (Sigma) and appropriate primers in StepOnePlus Real Time PCR System (Applied Biosystems). Beta-actin was used as an endogenous control for normalization. Primer sequences for the following genes:
ACTB-rtF: CTCCATCCTGGCCTCGCTGT
ACTB-rtR: GCTGTCACCTTCACCGTTCC
CDC20-rtF: GACCACTCCTAGCAAACCTGG
CDC20-rtR: GGGCGTCTGGCTGTTTTCA
ETV3-rtF: GGTGGAGGGTATCAGTTTCCT
ETV3-rtR TGATGAATGGGTAGTTGGGCAT
ELK1-rtF TCCCTGCTTCCTACGCATACA
ELK1-rtR GCTGCCACTGGATGGAAACT
ELK3-rtF ATCTGCTGGACCTCGAACGA
ELK3-rtR TTCTGCCCGATCACCTTCTTG
ELK4-rtF ACTCAGCCGAGCCCTCAG
ELK4-rtR GGTGGCTTTTTGGAAGGTG

EFR-rtF GCAAGCCCCAGATGAATTACG
EFR-rtR CCCCTTGGTCTTGTGCAGAA
ETV6-rt-F AGGCCATCCGTGGATAATGTG
ETV6-rt-R CGGTGATTTGTCGTGATAGGTGA

Cell culture and DNA damage induction

M14, HEK293, 7721, A375 cells were purchased from American Type Culture Collection and cultured in DMEM supplemented with 10% FBS and 1% penicillin and streptomycin and maintained in an atmosphere of 5% CO2 at 37 °C. Transient transfections were done with various expression plasmids in different cell lines using Lipofectamine 2000 (Invitrogen). According to manufacturer's protocol and cells were harvested after 48 h. For DNA damage induction, cells were treated with 1 mg/ml of 5-fluoro uracil (5FU) (Sigma).

Chromatin immunoprecipitation

ChIP was performed as described previously (Liu et al., 2014; Nelson et al., 2006). Briefly, protein–DNA complexes were cross-linked for 10 min at room temperature with 1% formaldehyde added directly into the culture medium. The reaction was stopped by the addition of glycine (final concentration 0.125 mol/L) and incubated for 5 min with gentle rocking. The cells were washed with PBS and buffer (10 mM Tris at pH 8.0, 10 mM EDTA, 0.5 mM EGTA, 0.25% Triton-X-100), suspended in 200 mL of lysis buffer (1.1% Triton- X-100, 4 mM EDTA, 40 mM Tris at pH 8.1, 300mM NaCl), and submitted to sonication to produce small DNA fragments (200–1000 base pairs). Chromatin was precleared and immunoprecipitated with the anti-flag M2 beads (Sigma). Precipitated DNA and protein complexes were reverse-cross-linked, and DNA fragments were purified with a QIAquick PCR purification kit (Qiagen). The purified DNAs were quantified by real-time Q-PCR. Primers to quantify the abundance of human CDC20 promoter were as follows:

CDC20-chipF TCACATCTTTAAAGCCCCAA
CDC20-chipR GTTTTACAAACAGGGAAAAT

Lentiviral shRNA-mediated knockdown

Plasmids expressing shRNA were constructed by cloning double strand oligonucleotides into the pLKO.1 vector containing the puromycin resistance gene. Lentiviral shRNA-mediated knockdown was performed as described previously (Liu et al., 2014). The oligonucleotides used for shRNA are listed

below:
ELK1-F
CCGGCCCAAGAGTAACTCTCATTATCTCGAGATAATGAGAGTTACTCT TGGGTTTTTTGGTACC
ELK1-R AATTGGTACCAAAAAACCCAAGAGTAACTCTCATT ATCTCGAGATAATGAGAGTTACTCTTGGG
ETV6-F
CCGGCCATAAGAACAGAACAAACATCTCGAGATGTTTGTTCTGTTCTT ATGGTTTTTTTGGTACC
ETV6-R
AATTGGTACCAAAAAACCATAAGAACAGAACAAACATCTCGAGATGTT TGTTCTGTTCTTATGGT
ETV3-F
CCGGCCTCAGATACTATTACAACAACTCGAGTTGTTGTAATAGTATCTG AGGTTTTTTGGTACC
ETV3-R
AATTGGTACCAAAAAACCTCAGATACTATTACAACAACTCGAGTTGTTG TAATAGTATCTGAGG
ERF-F
CCGGGAGGTGACTGACATCAGTGATCTCGAGATCACTGATGTCAGTC ACCTCTTTTTTGGTACC
ERF-R
AATTGGTACCAAAAAAGAGGTGACTGACATCAGTGATCTCGAGATCAC TGATGTCAGTCACCTC
ELK4-F
CCGGGCCCAAGTATTTCTCCATCTTCTCGAGAAGATGGAGAAATACTT GGGCTTTTTTGGTACC
ELK4-R
AATTGGTACCAAAAAAGCCCAAGTATTTCTCCATCTTCTCGAGAAGAT GGAGAAATACTTGGGC
ELK3-F
CCGGCTCCTCTTTAATGTTGCCAAACTCGAGTTTGGCAACATTAAAGA GGAGTTTTTTGGTACC
ELK3-R
AATTGGTACCAAAAAACTCCTCTTTAATGTTGCCAAACTCGAGTTTGG CAACATTAAAGAGGAG

Electrophoretic mobility shift assay (EMSA)

EMSA was performed using a chemiluminescent EMSA kit from Beyotime

Biotechnology following the manufacturer's instructions. Briefly, M14 cell nuclear extracts were prepared using NE-PER nuclear and cytoplasmic extraction reagents (ThermoFisher Scientific) according to the manufacturer's protocol. EMSA reactions included 1× binding buffer, 50 ng poly(dI-dC), 2.5% glycerol, 0.06% Nonidet P-40, 5 mM MgCl2, 19 μ g BSA, 2 μ l nuclear extract, and 20 fM biotin-labelled probes. Specificity of mobility shifts was analyzed by including un-labelled *CDC20* competitor oligonucleotides at the concentration of 8 pM. Reactions were incubated for 20 min at room temperature, size-separated on a 6% DNA retardation gel, and transferred to nylon membrane. Free or protein-bound biotin-labelled probes were detected using streptavidin–horseradish peroxidase conjugates and chemiluminescent substrate according to the manufacturer's protocol. Probe sequences for promoter regions are listed below:

WT F-5' ACTTTCCCCGGAAGGCCCGCCCCCT3'
R-5'AGGGGGCGGGCCTTCCGGGGAAAGT3'
525 F-5' ACTTTCCCCGAAAGGCCCGCCCCCT3'
R-5' AGGGGGCGGGCCTTTCGGGGAAAGT3'
528 F-5' ACTTTCCCCGGAAAGCCCGCCCCCT3'
R-5'AGGGGGCGGGCTTTCCGGGGAAAGT3'
529 F-5' ACTTTCCCCGGAAGACCCGCCCCCT3'
R-5'AGGGGGCGGGTCTTCCGGGGAAAGT3'

Supplemental References

Consortium, E.P. (2012). An integrated encyclopedia of DNA elements in the human genome. Nature *489*, 57-74.

Genomes Project, C., Auton, A., Brooks, L.D., Durbin, R.M., Garrison, E.P., Kang, H.M., Korbel, J.O., Marchini, J.L., McCarthy, S., McVean, G.A.*, et al.* (2015). A global reference for human genetic variation. Nature *526*, 68-74.

Hong, Y. (2013). On computing the distribution function for the Poisson binomial distribution. In Computational Statistics and Data Analysis, pp. 41-51.

Liu, X.S., Haines, J.E., Mehanna, E.K., Genet, M.D., Ben-Sahra, I., Asara, J.M., Manning, B.D., and Yuan, Z.M. (2014). ZBTB7A acts as a tumor suppressor through the transcriptional repression of glycolysis. Genes Dev *28*, 1917-1928.

Nelson, J.D., Denisenko, O., and Bomsztyk, K. (2006). Protocol for the fast chromatin immunoprecipitation (ChIP) method. Nat Protoc *1*, 179-185.

Roadmap Epigenomics, C., Kundaje, A., Meuleman, W., Ernst, J., Bilenky, M., Yen, A., Heravi-Moussavi, A., Kheradpour, P., Zhang, Z., Wang, J.*, et al.* (2015). Integrative analysis of 111 reference human epigenomes. Nature *518*, 317-330.

Siepel, A., Bejerano, G., Pedersen, J.S., Hinrichs, A.S., Hou, M., Rosenbloom, K., Clawson, H., Spieth, J., Hillier, L.W., Richards, S.*, et al.* (2005). Evolutionarily conserved elements in vertebrate, insect, worm, and yeast genomes. Genome Res *15*, 1034-1050.
